# The Genomic Basis of Adaptation to High Elevations in Africanized Honey Bees

**DOI:** 10.1093/gbe/evad157

**Published:** 2023-08-25

**Authors:** Turid Everitt, Andreas Wallberg, Matthew J Christmas, Anna Olsson, Wolfgang Hoffmann, Peter Neumann, Matthew T Webster

**Affiliations:** Department Medical Biochemistry and Microbiology, Science for Life Laboratory, Uppsala University, Uppsala, Sweden; Department Medical Biochemistry and Microbiology, Science for Life Laboratory, Uppsala University, Uppsala, Sweden; Department Medical Biochemistry and Microbiology, Science for Life Laboratory, Uppsala University, Uppsala, Sweden; Department Medical Biochemistry and Microbiology, Science for Life Laboratory, Uppsala University, Uppsala, Sweden; Grupo de Biocalorimetría, Universidad de Pamplona, Pamplona, Colombia; Institute of Bee Health, Vetsuisse Faculty, University of Bern and Agroscope, Bern, Switzerland; Department Medical Biochemistry and Microbiology, Science for Life Laboratory, Uppsala University, Uppsala, Sweden

**Keywords:** local adaptation, honey bee, admixture, introgression, natural selection

## Abstract

A range of different genetic architectures underpin local adaptation in nature. Honey bees (*Apis mellifera*) in the Eastern African Mountains harbor high frequencies of two chromosomal inversions that likely govern adaptation to this high-elevation habitat. In the Americas, honey bees are hybrids of European and African ancestries and adaptation to latitudinal variation in climate correlates with the proportion of these ancestries across the genome. It is unknown which, if either, of these forms of genetic variation governs adaptation in honey bees living at high elevations in the Americas. Here, we performed whole-genome sequencing of 29 honey bees from both high- and low-elevation populations in Colombia. Analysis of genetic ancestry indicated that both populations were predominantly of African ancestry, but the East African inversions were not detected. However, individuals in the higher elevation population had significantly higher proportions of European ancestry, likely reflecting local adaptation. Several genomic regions exhibited particularly high differentiation between highland and lowland bees, containing candidate loci for local adaptation. Genes that were highly differentiated between highland and lowland populations were enriched for functions related to reproduction and sperm competition. Furthermore, variation in levels of European ancestry across the genome was correlated between populations of honey bees in the highland population and populations at higher latitudes in South America. The results are consistent with the hypothesis that adaptation to both latitude and elevation in these hybrid honey bees are mediated by variation in ancestry at many loci across the genome.

**Significance**

Populations of honey bees in the Americas are hybrids of African and European ancestry in which local adaptation to climate appears to be governed by the relative proportion of these ancestries in the genome. Here, we present evidence that adaptation to high elevations in such hybrids in South America has a similar genetic basis. This indicates a polygenic basis for adaptation to both latitudinal and elevation clines.

## Introduction

Understanding the genetic basis of local adaptation is a key goal in evolutionary genomics, which is important for the conservation of wild populations in the face of climate change (Savolainen et al. 2013; Waldvogel et al. 2020; Hohenlohe et al. 2021; Webster et al. 2023). Many different forms of genetic variation underlie local adaptation. In some cases, one or a few loci with large effects govern adaptation to the local environment. For example, studies of adaptation to different environments in three-spined stickleback (Jones et al. 2012), adaptation to predation in deer and beach mice (Linnen et al. 2009; Steiner et al. 2009), and migratory behavior in cod (Berg et al. 2016) have revealed genetic variants with large phenotypic effect that govern local adaptation. Chromosomal inversions are commonly involved in local adaptation, as they have the possibility to fix multiple adaptive mutations on the same haplotype (Kirkpatrick and Barton 2006; Gutiérrez-Valencia et al. 2021). Many examples of adaptations controlled by inversions have been discovered in natural populations (Wellenreuther and Bernatchez 2018), such as a >100 Mb inversion in quails that influences multiple aspects of morphology and behavior (Sanchez-Donoso et al. 2022). However, in many other cases, local adaptation likely has a polygenic basis and can involve selection of many adaptive alleles of small effect distributed throughout the genome (Barghi et al. 2020; Tyrmi et al. 2020).

Adaptations with the same genetic basis can appear in geographically separated populations, or different species (Tiffin and Ross-Ibarra 2014). Studying such evolutionary convergence is an important way to understand the evolutionary constraints that govern adaptation (Stern 2013). For example, the *Ectodysplasin* locus in three-spined sticklebacks appears to govern adaptation to freshwater habitats in geographically distant locations (Jones et al. 2012). Adaptation to latitudinal climate variation in *Drosophila* spp. involves clinal variation in similar sets of loci across the northern and southern hemispheres (Adrion et al. 2015). Parallel local adaptation along clines is also seen in different populations of house mice (Ferris et al. 2021) and different species of conifer (Yeaman et al. 2016). It is unclear how common parallel local adaptation is, and whether it usually reflects the persistence of adaptive alleles across populations or new mutations.

The western honey bee, *Apis mellifera*, is native to Africa and Eurasia (Ruttner 1988) and has been introduced across the whole world, showing local adaptation to a wide range of environments (Wallberg et al. 2014). The genetic ancestry of honey bees has been apportioned into at least four divergent lineages that mirror morphometric analysis: A, C, M, and O (Ruttner 1988; Whitfield et al. 2006; Wallberg et al. 2014). Honey bees in Africa belong predominantly to the A group and exhibit a range of biological adaptations to habitats in lower latitudes. The majority of bees in Europe belong to the C and M groups in the east and west, respectively, and exhibit adaptation to temperate climates.

Genetic adaptations to high elevations have been uncovered in East African populations, governed by two chromosomal inversions on chromosomes 7 and 9, hundreds of kilobases in length (Wallberg et al. 2017). The inversions are found on three disconnected mountains in Kenya but have not been detected elsewhere in the world (Christmas et al. 2019). The functional effects of these inversions are not known, but it has been hypothesized that variation in the octopamine genes residing in the inversion on chromosome 7 could influence foraging behavior (Wallberg et al. 2017). The inversions are inferred to be ancient, and likely occurred in the range of 1–4 million years ago, suggesting they arose before the dispersal of honey bees over the world (Wallberg et al. 2017, 2014). Although honey bees are present in high-altitude locations in many parts of the world, the degree to which they possess specific adaptations and whether the same inversions are also present are unknown.

Honey bees are not native to the Americas, but were imported by European settlers from the 1,600 s onwards (Crane 1999). The genetic ancestry of these honey bees was likely typical of those in Europe (subspecies belonging to the M and C groups). However, in 1957, swarms of African ancestry honey bees were accidently released from São Paulo in Brazil (likely *A. m. scutellata* from the A group). This led to a massive biological invasion, whereby the existing honey bees were replaced by those with mainly African ancestry, commonly referred to as “Africanized” bees (Winston 2014). Africanized bees expanded into their southern limits in Argentina in the 1960s and reached the southern USA in the 1990s.

Stable hybrid zones now exist between populations of honey bees of predominantly European and African ancestry, which are found at highly similar latitudes in both North and South America, representing the limits of the distribution of Africanized bees (Calfee et al. 2020). Investigations of genetic ancestry are consistent with the idea that tropical and subtropical regions bounded by these hybrid zones are now predominantly populated by honey bees of high African ancestry (Rinderer et al. 1991; Sheppard et al. 1991; Clarke et al. 2002; Pinto et al. 2005; Rangel et al. 2016). Genome sequencing of populations from across Brazil showed that 84% of their ancestry can be attributed to the A group, with the remaining part from the M group (Nelson et al. 2017), a situation that is likely fairly homogenous throughout the tropics.

Across the American hybrid zones, there is a gradual decrease in A group ancestry in favor of both M and C ancestry with increasing distance from the equator, which is mirrored by an increase in forewing length with latitude in these zones (Calfee et al. 2020). The steepness of the ancestry clines varies across the genome but is not focused on specific loci. However, cline steepness at loci across the genome is correlated in the hybrid zones in the northern and southern hemispheres, indicating that the strength of selection varies across the genome in a repeatable way, targeting a similar set of loci in essentially unconnected populations. These observations suggest that adaptation to climate is polygenic and mediated by ancestry proportions at many loci across the genome.

Populations of honey bees are found at high elevations in several locations in the Americas (Quezada-Euán et al. 2003; Winston 2014; Orjuela Parrado 2018). However, little is known about the existence of adaptations to these habitats or their genetic basis. A study of variation in genetic ancestry in Peru found that A group ancestry declined with elevation and was found at lower proportions at elevations above 1,500 m (Quezada-Euán et al. 2003). Africanized honey bees have been present in Colombia since at least 1980, suggesting that the type A ancestry in honey bees has reached an equilibrium. A study of morphometrics of bees in Colombia found a positive correlation between forewing length and elevation, similar to the trend across latitudes, with bees at higher elevations having significantly longer forewings (Orjuela Parrado 2018). Hence similar genetic and morphological changes appear to occur with both latitude and elevation.

Here, we sequenced 29 whole genomes of honey bee workers, each collected from different colonies located in high and low elevations in the Norte de Santander region of Colombia, with the aim to uncover the genetic basis of local adaptation. We address two main hypotheses: First, are the genomic regions involved in high-elevation adaptation in East Africa also under selection at high elevations in Colombia (Christmas et al. 2019)? Second, is there evidence for ancestry-mediated polygenic adaptation to high elevations as observed over latitudinal clines of hybrid bees in the Americas (Calfee et al. 2020)?

## Results

### Genetic Variation in Colombian Honey Bees

**Fig. 1.**
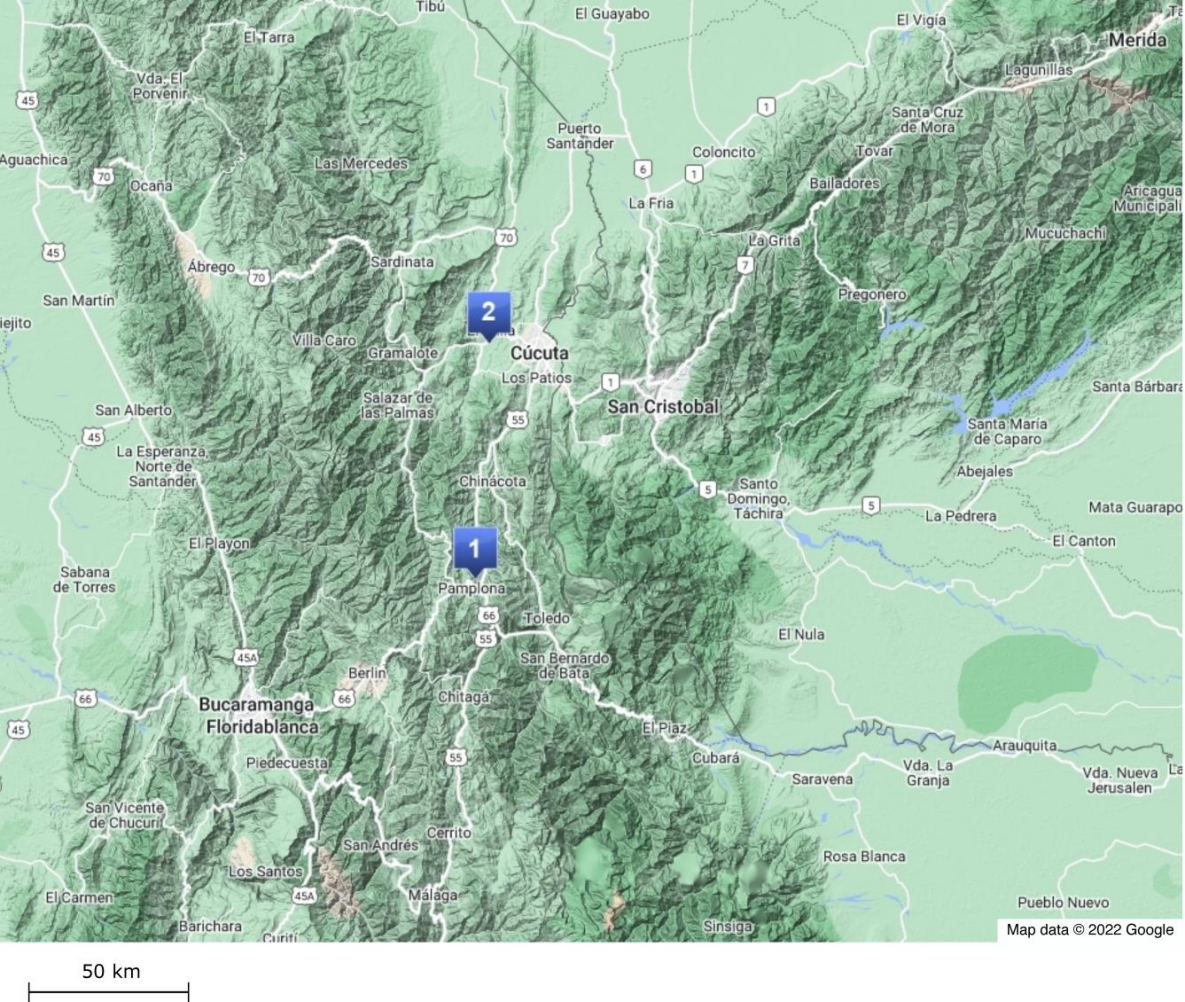
Sampling locations for Africanized honey bee colonies. 1 = location for highland bees (Pamplona, 2,400 m asl); 2 = location for lowland bees (Carmen de Tonchalá, 317 m asl).

We sequenced 15 honey bee workers from Carmen de Tonchalá (320 m above sea level, referred to as lowland bees) and 14 from Pamplona (2,420 m above sea level, referred to as highland bees) (fig. 1; supplementary table S1, Supplementary Material online). Each sample came from a different colony. The average read depth per sample was 10.5 × . In addition, we obtained data for three reference populations (19 Kenyan worker bees from the A group, 9 East European worker bees from the C group, and 85 Iberian drones from the M group) from published studies (Harpur et al. 2014; Wallberg et al. 2017; Henriques et al. 2018; Christmas et al. 2019). The Kenyan worker bees came from the lowland population used in previous studies of mountain bees (Wallberg et al. 2017; Christmas et al. 2019). All samples were genotyped together producing a dataset of 5.4 million single nucleotide polymorphisms (SNPs) after quality filtering (see Materials and Methods). Levels of genetic variation found in each of the populations are shown in table 1. In agreement with previous results, the Kenyan population has a higher nucleotide diversity than the other two non-African reference populations, and the hybrid Africanized populations also have high levels of genetic variation (Wallberg et al. 2014).

**Table 1:** Genome-Wide Estimates of Nucleotide Diversity

Population	Group	Subspecies	No. Genomes	No. SNPs	Nucleotide Diversity (π)
Kenyan	A	*scutellata*	38	4,804,181	0.00439
East European	C	*carnica*	18	1,295,086	0.00145
Iberian	M	*iberiensis*	84	2,172,565	0.00173
Colombian HL	Admixed	Hybrid	28	4,042,961	0.00418
Colombian LL	Admixed	Hybrid	30	4,141,402	0.00397

**Fig. 2.**
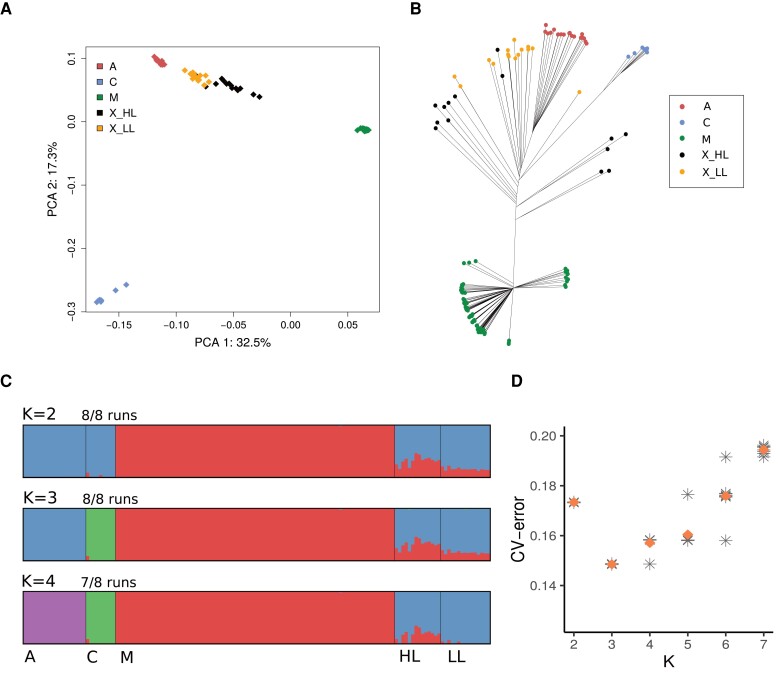
*A*) PCA-plot and *B*) neighbor-joining tree based on 372,205 SNPs. The samples are colored by ancestry type: A = Kenyan bees from the A group; C = East European bees from the C group; M = Iberian bees from the M group; X_HL = Colombian highland bees (mixed ancestry); X_LL = Colombian lowland bees (mixed ancestry). *C*) Genome-wide ancestries per individual for different numbers of ancestral populations (*K*). The major mode from eight different runs of ADMIXTURE, identified with pong (Behr et al. 2016) is shown for each value of K. For *K* = 2 and *K* = 3, the results from all runs support the same mode but for higher values of K, multiple modes appear in the results. D) CV errors for eight different runs of ADMIXTURE and different values of K. Diamonds show the mean CV-error for each K-value.

We estimated population structure in the samples using principle component analysis (PCA) and a phylogenetic tree using a thinned set of 372,205 SNPs (see Materials and Methods). In the PCA plot (fig. 2*A*), the first and second components (which explain 32.5% and 17.3% of the variation, respectively) clearly separate the samples into different clusters representing the A, C, and M groups (Wallberg et al. 2014). The Colombian samples are positioned approximately on a line between the A and M clusters, with the lowland samples closer than most of the highland samples to the A cluster. A similar pattern is seen in the neighbor-joining tree in figure 2*B*, where the A, C, and M groups all form their own distinct clusters. The Colombian samples appear between the A and M groups in the tree, with the lowland samples clustering closer to the A group. None of the Colombian samples are close to the C group, which separates from the other samples on a long branch. These two analyses both indicate that the Colombian samples are a mixture of A and M ancestries, with a lack of contribution from the C group.

We assessed the highland and lowland Colombian samples for evidence of population substructure and the presence of related samples. A PCA plot of these samples does not indicate the presence of substructure or clusters of samples with elevated relatedness (supplementary fig. S1, Supplementary Material online). In addition, we scanned all pairwise combinations of samples for elevated kinship coefficients (Manichaikul et al. 2010). This coefficient does not exceed the threshold needed to infer any degree of relatedness in any of the sample pairs (0.044). We therefore consider all of the samples to be unrelated.

### Highland Honey Bees Have a Greater Proportion of European Ancestry

Genome-wide ancestry estimation was performed using the software ADMIXTURE (Alexander et al. 2009). The assumed number of ancestral populations, K, was varied between two and seven and this was repeated in eight independent runs with different seeds. The cross-validation (CV) error based on 10-fold CV was lowest for K = 3 in each of the runs, indicating this to be the optimal number of ancestral populations. The major modes of the clustering results were identified with the software pong (Behr et al. 2016) and are shown in figure 2*C*. At *K* = 3, the A, C, and M groups separate into different clusters while the Colombian samples appear as a mixture of the A and M ancestries, with only around 0.1% contribution from the C groups. The mean A ancestry proportion of the Colombian lowland samples is 84.7%, which is identical to samples collected across Brazil (Nelson et al. 2017) and typical for honey bees in this tropical/subtropical region of the world. In the Colombian highland samples, the proportion of A ancestry is significantly lower, at 68.6% (Welch's two-sided *t*-test, *P* < 0.0001). This change in ancestry with elevation resembles the clinal ancestry changes with latitude observed in both North and South America, where the type A ancestry decreases further away from the equator (Calfee et al. 2020).

### Signals of Ancestry-mediated Selection in the Genomes of Highland Honey Bees

**Fig. 3.**
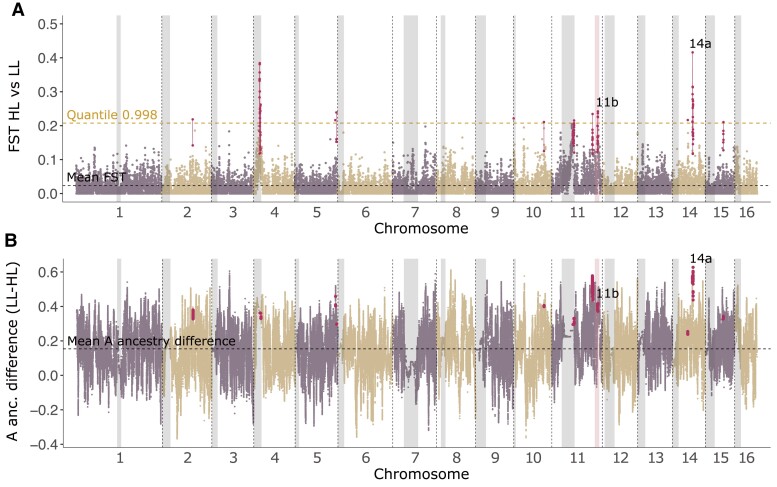
*A*) *F_ST_* of Colombian highland population versus Colombian lowland population estimated in 10 kbp windows. Shaded grey areas mark the putative pericentromeric regions. The shaded pink region on chromosome 11 denotes a region previously identified by Nelson et al. (2017). The quantile corresponding to 99.8% of the *F_ST_* -values is shown as a dashed yellow line. *F_ST_* peaks colored in maroon fulfill the criteria described in Materials and Methods and are described in more detail in supplementary tables S2 and S3, Supplementary Material online. *B*) Mean probability of A ancestry per SNP in lowland population minus the mean probability of A ancestry per SNP in highland population. The *F_ST_*-peak regions are also marked in the ancestry plots for comparison.

We scanned the genome for signals of selection by performing multiple pairwise *F_ST_* comparisons between populations using 10-kbp windows and by estimating variation in ancestry proportions across the genome in the Colombian honey bees. We first compared the Colombian highland and lowland populations (fig. 3*A*). We did not observe any regions of elevated *F_ST_* in the vicinity of the two chromosomal inversions on chromosomes 7 and 9 that were identified in the Kenyan highland populations (Wallberg et al. 2017; Christmas et al. 2019). This strongly suggests that variation in these regions does not underlie adaptation in the Colombian populations studied here. It is unknown whether the two inversions are present in either of these populations.

We used coalescent simulations to generate an expected background pattern of allelic divergence under neutral genetic drift. We tested for signatures of excess divergence between Colombian highland and lowland bees compared to neutrality, which could indicate adaptive divergence driven by natural selection between environments. To this end, we pruned the thinned SNP dataset (372,205 SNPs) into the subset of 248,598 SNPs that were polymorphic in the Colombian bees (on average, 906 bp per SNP), and generated a per-SNP *F*_ST_ distribution from them. We then generated a simulated *F*_ST_ distribution for this number of SNPs. The two sets of SNPs had very similar average *F*_ST_ values (empirical = 0.058 vs. simulated = 0.055), indicating a similar level of average divergence.

We considered windows greater than the 0.998 quantile of *F*_ST_ as outliers. Above this threshold (*F*_ST_ > 0.255), we observe 3,164 SNPs in our empirical dataset but only 2,442 SNPs in the simulated dataset, a 30% enrichment of highly divergent SNPs compared to what would be expected from neutral evolution alone. Hence there is evidence that selection has resulted in high *F*_ST_ values of a subset of SNPs. These highly divergent SNPs are apportioned into 14 distinct peaks (fig. 3*A*). The peak with the highest *F*_ST_ window spans 30 genes on chromosome 14 (labeled 14a). Another prominent peak (labeled 11b) overlaps a region of significantly reduced African ancestry in South American bees identified in two previous studies (Nelson et al. 2017; Calfee et al. 2020).

We investigated differences in the proportion of A ancestry across the genome in the highland and lowland samples (fig. 3*B*). There is a highly significant correlation between *F*_ST_ and A ancestry difference between the lowland and highland samples (Spearman's *ρ* = 0.26, *P* < 10^−16^). This indicates that the most divergent loci are associated with the largest drops in A ancestry in the highland population. Among *F*_ST_ outliers, the mean drop in A ancestry in the highland population is 0.4843 compared to a genome-wide average of 0.1538 and all of these outliers show a greater decrease in A ancestry than average.

**Fig. 4.**
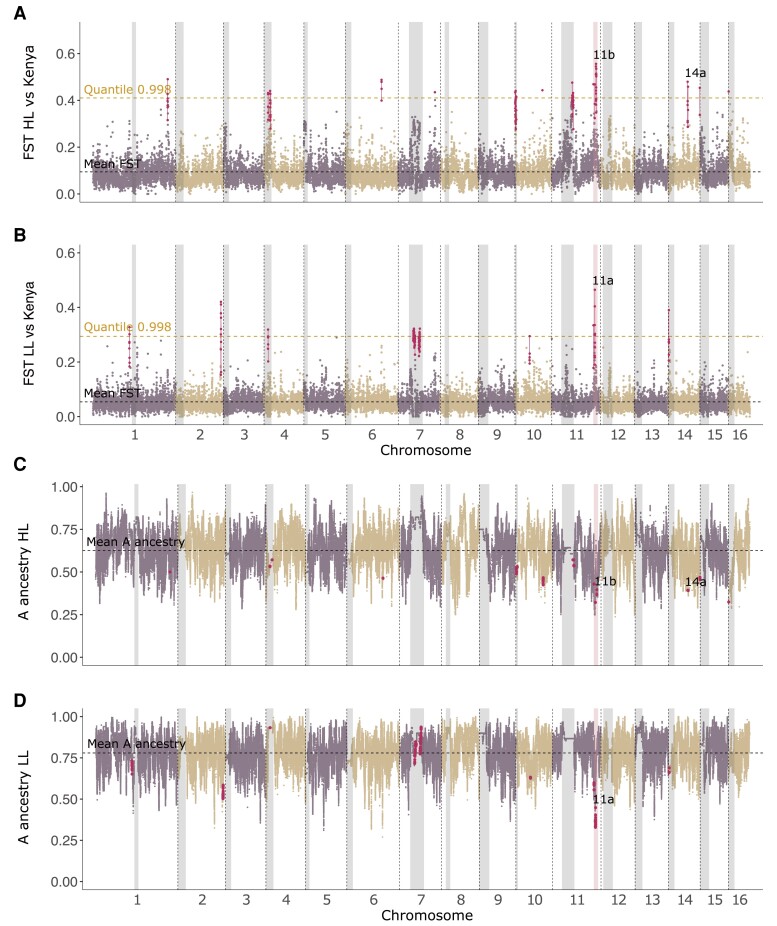
*A*) *F_ST_* of Colombian highland population versus Kenyan samples. *B*) *F_ST_* of Colombian lowland population versus Kenyan samples. *C*) Mean probability of ancestry from the A group per SNP in Colombian highland population. *D*) Mean probability of ancestry from the A group per SNP in Colombian lowland population. *F_ST_* is estimated in 10 kbp windows. Shaded gray areas mark the putative pericentromeric regions. The shaded pink region on chromosome 11 denotes a region previously identified by Nelson et al. (2017). The quantile corresponding to 99.8% of the *F_ST_* -values is shown as a dashed yellow line. *F_ST_* -peaks colored in maroon fulfill the criteria described in Materials and Methods and are described in more detail in supplementary tables S2 and S3, Supplementary Material online. *F_ST_* -peaks are also marked in the ancestry plots for comparison.

We next performed *F_ST_*-scans comparing the two Colombian populations separately to the Kenyan population in order to identify regions that differ from the ancestral African progenitors, with the aim of uncovering the origin of differences between highland and lowland populations. The *F_ST_* scan between the Colombian highland population and the Kenyan population (fig. 4*A*) revealed several regions of the genome in which *F_ST_* exceeds the 0.998 quantiles. The majority of these regions correspond to *F*_ST_ outliers in the previous highland-lowland comparison, indicating that they are candidates for involvement in adaptation to highlands. The two most prominent of these regions are peak 11b (14.8–15 Mbp on chromosome 11) and peak 14a (6.5–6.56 Mbp on chromosome 14) which were also identified in the highland-lowland comparison. A third prominent region, peak 11a (14.43–14.53 Mbp on chromosome 11), is most pronounced in the *F_ST_* comparison between the lowland population and the Kenyan population and is not present in the highland-lowland comparison (fig. 4*B*; supplementary table S2, Supplementary Material online). It has reduced type A ancestry in both the lowland and the highland population (fig. 4*C* and *D*). Detailed plots of peaks 11a, 11b, and 14a in all *F_ST_* comparisons are shown in supplementary figures S1–S6, Supplementary Material online. The coordinates and *F_ST_* values of all peaks and the genes within them are summarized in supplementary tables S2 and S3, Supplementary Material online.

The peaks 11a and 11b both overlap a region on chromosome 11 that has previously been identified by Nelson et al. (2017) and Calfee et al. (2020). In the study by Nelson et al., a region of low ancestry from the A group was identified in Africanized honey bees from Brazil. The coordinates of this region, identified through Basic Local Alignment Search Tool (BLAST) alignment to the latest version of the reference genome (Amel_Hav3.1), are 14.0–15.4 Mbp. This region overlaps quantitative trait loci’s (QTLs) related to foraging behavior, ovary size, and ovariole number identified in previous studies (Linksvayer et al. 2009; Rueppell 2009; Ihle et al. 2015) as well as the *mTOR* gene (mechanistic target of rapamycin; LOC409393), whose product serine/threonine protein kinase is involved in multiple processes in the cell and affects the caste differentiation of female bees (Mutti et al. 2011). It is hypothesized that the European forms of those traits have selective advantages in the neotropics (Nelson et al. 2017). Calfee et al. (2020) identified a similar region (13.9–15.3 Mbp on chromosome 11; Amel_HAv3.1) where Africanized honey bees from Argentina have reduced ancestry from the A group, but this pattern was not present in a sample of Africanized bees from California.

Our results are consistent with previous studies indicating that a region on chromosome 11 overlapping peaks 11a and 11b has elevated A type ancestry in South America (Nelson et al. 2017; Calfee et al. 2020). They also provide suggestive evidence that selection at loci within this region (particularly peak 11b) could modulate adaptation to higher elevations, as allele frequencies in the highland and lowland populations diverge in this region. In addition to this, peak 14a on chromosome 14, and several other regions, show pronounced divergence between the highland and lowland populations indicating that they may contain genetic variants underlying adaptation to elevation.

The *F_ST_* comparisons also revealed a number of peaks with elevated *F_ST_* that were inferred to be in repetitive pericentromeric regions, which are associated with low recombination rates (figs. 3 and 4). A range of studies including those in honey bees and bumblebees have identified excess divergence between populations in such regions (Henikoff and Malik 2002; Ellegren et al. 2012; Cruickshank and Hahn 2014; Booker et al. 2020; Parejo et al. 2020; Christmas et al. 2021). Prominent *F_ST_* peaks outside of centromeric regions also have reduced recombination rates: For peaks 11a and 11b, the recombination rates are 39% and 10% of the chromosomal average, respectively, whereas the recombination rate in peak 14a, is 41% of the chromosomal average.

### Enrichment of Genes With Functions in Reproduction

We computed gene-wide *F*_ST_ values for 11,775 genes (supplementary table S5, Supplementary Material online) and interrogated the most divergent genes for shared gene ontologies (GOs) that could inform about potential biological functions being under selection. Fourteen genes had *F*_ST_ values >0.255 (the 0.998 quantiles based on all SNPs) and were tested for GO enrichment compared to all genes using *Drosophila* homologs. Among these candidates, we detected strong enrichment (false discovery rate <0.05) for genes involved in fatty acid metabolism, meiosis, and sexual reproduction (supplementary table S6, Supplementary Material online), including honey bee homologs of three genes implicated in mating behavior (e.g., *Desat1, CG9997, bol*). The three candidates represent unlinked loci with high allelic divergence located on three different chromosomes (4, 11, 14), including peak 14a. Two of them have roles in sperm function such as spermatogenesis (e.g., *bol*) or sperm competition (e.g., *CG9997*).

### Genome-Wide Ancestry Correlations

In the study by Calfee et al. (2020), changes in type A ancestry along latitudinal transects in Argentina and California were modeled as logistic clines for each SNP. The cline steepness for each SNP can be used as a measure of the rate of ancestry change. We investigated if there was a correlation between the cline steepness across Argentina and changes in ancestry proportions between our Colombian highland and lowland populations at the same SNPs. The Californian clines were not included in this comparison as the Californian populations all have lower A ancestries than the Colombian populations. The genome-wide Pearson correlation between the ancestry changes in Colombia and Argentina was low (*r* = 0.011) but highly significant (*P* < 10^−11^).

**Fig. 5.**
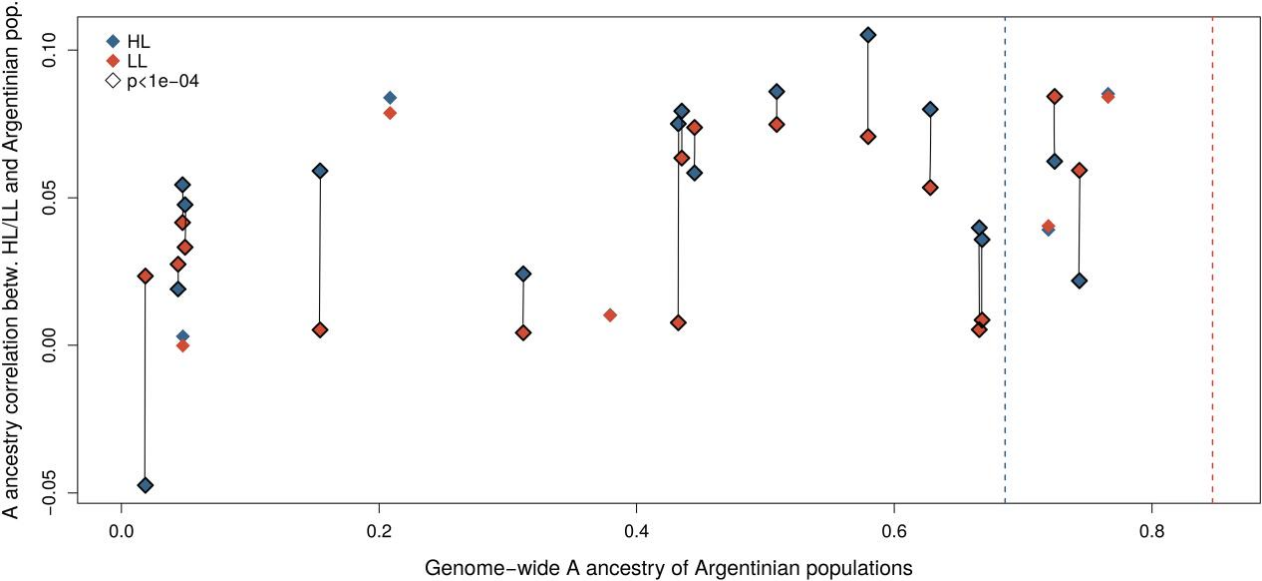
Pearson correlations of ancestry probabilities from the A group per site across the genome, between each of the Argentinian populations and the Colombian highland population (HL), and the Colombian lowland population (LL). The correlation coefficients (y-axis) are plotted against the genome-wide A ancestry of the Argentinian populations on the x-axis, as estimated with the ADMIXTURE software. The dashed vertical lines show the genome-wide A ancestry of the Colombian highland population (HL) and Colombian lowland population (LL). For Argentinian populations where the correlation to the Colombian highland population is highly significantly different (*P* < 0.0001) from the correlation to the Colombian lowland population, the data points are marked with a black border around the diamond. Vertical lines connect comparisons of the highland (HL) and lowland (LL) Colombian samples with the same Argentinian sample.

We next wished to determine whether the proportion of A ancestries across the genome is more strongly correlated in populations inhabiting similar climates, which could suggest similar selective pressures. We measured the correlation of A ancestry probabilities across the genome between the two Colombian populations and each of the Argentinian populations separately (fig. 5). Among these populations, non-A ancestry comes predominantly from the M group for populations with A ancestry greater than 0.5, similar to the Colombian populations studied here (Fig. 1 in Calfee et al.). Out of 21 Argentinian populations in total, 17 have lower levels of A type ancestry than the highland Colombian population, whereas 4 have levels of A type ancestry at levels between the highland and lowland populations. Eleven of the 17 populations in the first class show significantly higher correlation with genome-wide ancestry in the highland compared to the lowland population (*P* < 0.001 significance cutoff; see Materials and Methods) whereas none of the four in the second class show significantly higher correlations with the highland population. This difference is statistically significant (*P* < 0.05; Fisher's exact test). Hence the highland population tends to show stronger genome-wide correlations in ancestry with populations at higher latitudes in Argentina, which experience relatively more similar climates despite large geographical separation. This observation is consistent with findings presented by Calfee et al. (2020), who found that variation in ancestries across the genome were correlated between populations in higher latitudes in Argentina and California. The results suggest that an overlapping suite of loci across the genome mediates adaptation to higher latitudes and higher elevation in hybrid honey bees in the Americas.

## Discussion

We compared genome variation in populations of Africanized honey bees from a highland and lowland location in Colombia in order to uncover the genetic basis of adaptation to high-elevation habitats in these populations. The main findings are 1) a significantly reduced level of African (A group) ancestry in the highland compared to the lowland population; 2) identification of genomic regions with elevated divergence between the two populations, which contain candidate genes for local adaptation to high elevations; 3) correlations in ancestry proportions across the genome between populations in similar habitats, indicating that similar sets of loci with small effects may influence local adaptation across latitudinal and elevational gradients.

Previous studies indicate that honey bees have a consistently high (>80%) A group ancestry throughout low-elevation localities in the tropics (Rinderer et al. 1991; Sheppard et al. 1991; Clarke et al. 2002; Pinto et al. 2005; Rangel et al. 2016; Nelson et al. 2017; Calfee et al. 2020). This transitions into hybrid zones in more temperate regions in both North and South America containing clines that span hundreds of kilometers where the proportion of A group ancestry approaches zero in colder climates, replaced by European (C and M group) ancestry (Calfee et al. 2020). Both Colombian populations studied here have predominantly African (A group) ancestry, which is consistent with other populations studied in the tropical South American region. A previous genomic analysis of honey bees sampled across Brazil found that 84% of their ancestry comes from the A group (Nelson et al. 2017), which is highly similar to the A ancestry proportion of 84.7% in the Colombian lowland population. The 32 samples from Brazil were all collected from low elevations, ranging from sea level to 900 m above sea level. There was minimal variation in average ancestry levels among samples, with the exception of two samples collected in the southernmost locality (Rio Grande do Sul) with slightly lower African ancestry (75%) (Nelson et al. 2017). This locality overlaps the hybrid zone studied by Calfee et al. (2020). In contrast, A group ancestry in the highland Colombian (68.6%) bees is lower than other samples collected from tropical regions.

After their introduction in 1957, the spread of Africanized bees in South America was initially resisted by the importation of queens of European origin. However, as Africanized bees readily take over managed colonies of European bees, this strategy was largely abandoned in favor of working with local Africanized bees (Winston 2014). Populations of honey bees across South America are therefore expected to have reached genetic equilibrium in terms of ancestry (Diniz et al. 2003; Branchiccela et al. 2014), which is consistent with the finding of an A ancestry close to 85% in all colonies sampled within the two hybrid zones in North and South America (Nelson et al. 2017; Calfee et al. 2020). Africanized honey bees have been present in Colombia for over 40 years and the vast majority of beekeepers in Colombia work with Africanized honey bees derived from locally captured feral swarms (Tibatá et al. 2018). Mating between managed colonies and numerous feral colonies occurs freely, and they are expected to have equivalent genetic ancestries. There also is limited movement of managed honey bee colonies in Colombia. Africanized honey bee colonies do not require special attention to thrive, as they are naturally resistant to *Varroa* mites, which pose significant risks to managed colonies with European ancestries in many other parts of the world (Tibatá et al. 2021).

A weakness of our study is the limited sampling, which included colonies from only one highland and one lowland location. However, considering the limited movement of managed colonies and the free breeding between feral and managed colonies, the samples we collected are expected to accurately reflect the genetic variability in each local region. The consistent differences in ancestry proportions between the two populations suggest that there is likely a stable ancestry cline with elevation in this region, mediated by a selective advantage of European ancestry at certain loci at higher elevations, similar to the ancestry clines observed with latitude in hybrid zones in North and South America (Calfee et al. 2020). We predict that similar ancestry differences are present in high-elevation populations of Africanized bees elsewhere in the Americas, but more extensive sampling is required to test this.

Previous analysis of honey bee adaptation to high elevations in East African mountains identified two large chromosomal inversions on chromosomes 7 and 9 present at high frequencies in highland populations (Wallberg et al. 2017; Christmas et al. 2019). One of these inversions contains a cluster of octopamine genes, which have subsequently been shown to be important in honey bee thermogenesis, making them strong candidates for involvement in climatic adaptation (Kaya-Zeeb et al. 2022). We do not observe elevated *F_ST_* in these regions in any of our population comparisons. As the population of African bees initially released in Brazil in 1957 was relatively small and did not contain bees from highlands (Winston 2014), it is likely that none of the original founders possessed this inversion. It is interesting to note that, despite its importance in adaptation to climate (Wallberg et al. 2017; Christmas et al. 2019), no signals of selection were detected in this genomic region in populations from different latitudes (Calfee et al. 2020) or elevations (this study) in the Americas.

Two prominent regions of elevated divergence between the highland and lowland Colombian populations were identified in this study (peaks 11b and 14a, fig. 3, supplementary figs. S2–S7, Supplementary Material online). These regions also differ between the highland population and the Kenyan population, indicating that the highland population is most divergent in this region. Furthermore, we identified a region of elevated divergence between the lowland population and the Kenyan population (peak 11a; fig. 4), which is not divergent in the highland population. Peaks 11b and 14a are therefore candidate regions for adaptation to high elevation, whereas peak 11a is not. Both peak 11a and 11b overlap a previously identified 1.4 Mbp region on chromosome 11 with reduced A ancestry in Argentinian and Brazilian honey bees (Nelson et al. 2017; Calfee et al. 2020). This region is particularly interesting as it contains QTLs associated with reproductive traits and foraging behavior (Linksvayer et al. 2009; Rueppell 2009; Ihle et al. 2015). It is therefore plausible that selection has acted on variation in this region both during the initial spread of Africanized bees in the Americas and their expansion into novel environments at high elevations.

Peak 11b contains 28 genes, including those involved in a variety of processes including mitosis, nicotinamide adenine dinucleotide phosphate generation, repression of retrotransposons, endocytic recycling, and cytoskeleton remodeling. It contains the *mTOR* gene (mechanistic target of rapamycin; LOC409393), involved in caste differentiation of female bees (Mutti et al. 2011), and the myeloid leukemia factor *MLF* (LOC409877). The latter is expressed in the crystal cells of the hemolymph in *Drosophila melanogaster* (Martin-Lannerée et al. 2006), which are involved in wound healing and innate immune responses (Evans et al. 2003). Further candidates for local adaptation to highlands are found in peak 14a on chromosome 14 which contains 30 genes. One of these genes is *Samui* (LOC727486), which has been shown to be important in cold defense in silkworm eggs (Moribe et al. 2001), although it is unclear whether this function would be important in honey bees that thermoregulate their colonies. Peak 11a contains 20 genes including transmembrane protein 64 (LOC413916), which regulates osteoblast and adipocyte differentiation in mice (Jeong et al. 2015); alanine-glyoxylate aminotransferase-2 (LOC100576186), which regulates nitric oxide (NO) levels and blood pressure in mice (Caplin et al. 2012); and Eleven nineteen Lysine rich Leukemia-associated factor, *Eaf* (LOC411433), which is involved in transcriptional regulation of *Hsp70* (heat-shock protein 70) and developmental genes through the activity of RNA polymerase II in human cells (Kong et al. 2005).

We used a coalescent simulation based on a simplified population history to model the divergence between the highland and lowland populations. Compared to this simulation we find a 30% enrichment of SNPs under selection above our cutoff of *F*_ST_ > 0.255 in our dataset. It is important to note that these simulations assume constant population size and do not explicitly model variation in recombination rate between loci. Both of these factors are likely to influence the variance in coalescent times and the null expectation of the distribution of *F_ST_* and hence our interpretation of the number of SNPs likely involved in local adaptation.

The enrichment of highly divergent genes with functions in sperm and reproduction could indicate that these functions are important for adaptation to highlands. Bees with African ancestry have a higher degree of polyandry which might indicate stronger selection due to sperm competition (Franck et al. 2000; Hernández-García et al. 2009). Alternatively, the high divergence could reflect that such genes are typically fast evolving and, therefore, more likely to be associated with selective sweeps (Civetta and Singh 1995). However, a study in *Drosophila* found that rapid evolution of a proportion of reproductive genes was likely attributable to relaxed selection rather than positive selection (Patlar et al. 2021). A similar effect could potentially contribute to the enrichment of these categories in our gene ontology analysis.

We also observe that genomic peaks of divergence between populations are often associated with regions inferred to be centromeric, which tend to have low recombination rates (Wallberg et al. 2019). Recurrent linked selection—either background selection or genetic hitchhiking—can lead to elevated divergence in regions of low recombination, although this does not exclude their involvement in local adaptation (Cruickshank and Hahn 2014). In addition, transmission biases such as centromere drive may contribute to elevated divergence in centromeric regions (Talbert and Henikoff 2022). Other prominent peak regions (11a, 11b, and 14a) also have reduced recombination rates, suggesting that linked selection may have contributed to their increased divergence.

In addition to outlier loci, the genome-wide ancestry proportions are significantly different between the highland and the lowland population. As there are no barriers to gene flow between the populations, which would homogenize ancestry proportions, this likely indicates that these differences are driven by selection and that adaptation to differences in elevation has a broad genetic basis involving a large number of low-effect loci. Calfee et al. (2020) studied clinal variation of ancestry with latitude at SNPs across the genome in the two hybrid zones in North and South America, finding that the clines had similar shapes at the majority of loci in the genome, with very few outliers, and that steeper clines were observed in regions of low recombination. This supports the hypothesis that adaptation to latitudinal differences in the environment is controlled by a large number of loci across the genome (Calfee et al. 2020). The steepness estimates of the clines across South America are significantly correlated to the ancestry changes between the highland and lowland populations in Colombia. This could indicate that a similar set of loci contributes to differences in ancestry between these two populations. However, the correlation is weak, which could indicate that the overlap is limited but could also be influenced by the fact that the transects in South America (Calfee et al. 2020) encompass samples with much lower A ancestries than those found in our samples in Colombia (fig. 5).

We also find that the highland Colombian population shows greater correlation of ancestry proportions across all SNPs with populations at higher latitudes in South America. A similar pattern was observed by Calfee et al. (2020), who found that ancestry proportions at SNPs across the genome were correlated between populations from similar habitats in North and South America, despite massive geographical separation. Our results are analogous to this observation and indicate that polygenic adaptation at an overlapping set of loci could govern adaptation to environmental variation with both latitude and elevation.

The selective forces that drive adaptation across latitudinal and elevational gradients are unknown, although it is clear that honey bees with predominantly African traits are unable to survive winter conditions. Climate could play an important role either directly due to a factor such as temperature or indirectly due to characteristic habitats presents in climate zones. Calfee et al. (2020) investigated whether any specific climate parameter could be responsible for the ancestry changes between the honey bee populations in North and South America, but did not find any better predictor than the latitude. A study conducted in Costa Rica found that Africanized colonies had higher survivorship than European ones, but that this difference was much less pronounced in highland locations (Spivak 1992).

The lowland location studied here has a warm and humid climate, with a mean annual temperature of 18–20 °C and 38–205 mm precipitation per month, while the highland location is cooler and drier, with 11–12 °C mean temperature and 14–99 mm precipitation per month (Zepner et al. 2021). The Colombian highland habitat sampled in this study belongs to Köppen climate class Cfb (oceanic climate; similar to Cfa but slightly cooler) while the Colombian lowland habitat belongs to climate class Af (tropical rainforest climate). The climate class of all the Argentinian sampling locations (Cfa) is also more similar to the Colombian highland location (Cfb) than to the Colombian lowland location (Af). Hence regions with similar climates tend to be inhabited by honey bees with similar ancestry proportions, but the main factor that drives adaptation is unknown.

Although we have not directly investigated morphology in this study, ancestry differences appear to be correlated with differences in morphology. A study of wing morphology in Colombian honey bees has reported an increase in forewing length with elevation, from 8.46 mm at 986 m above sea level to 9.41 mm at 2,621 m above sea level (Orjuela Parrado 2018), elevations similar to those studied here. Similar morphological differences are seen in mountain honey bees in East Africa (Radloff and Hepburn 2000; Gruber et al. 2013). A similar trend also has been identified with latitude in both North and South America, where the forewing length increases further away from the equator while the African ancestry decreases (Calfee et al. 2020). Across those hybrid zones, the wing lengths change by 0.5 mm on average. Hence the decreasing proportion of A ancestry is mirrored by increasing wing length along both latitudinal and elevational clines.

A trend of increasing wing length with latitude was also observed in California before this area was invaded by African hybrid bees (Daly et al. 1991), indicating that this morphological feature is important for environmental adaptation. This can be explained by a tradeoff between flight efficiency and thermoregulation, whereby larger bees are better able to preserve heat in order to survive cold temperatures, whereas smaller bees are more efficient at flying (Hepburn et al. 1999). Honey bees from African subspecies have smaller size and wing lengths than European bees, and analysis of the dimensions of flight machinery has shown that African bees are designed to fly more efficiently. These morphological differences are likely to be an important component of environmental adaptation.

This study highlights the importance of hybridization as a factor driving local adaptation, due to an influx of alleles beneficial to the recipient population (Hedrick 2013). Hybridization has facilitated evolution in adaptive radiations such as Darwin finches (Lamichhaney et al. 2015), and *Heliconius* butterflies (The Heliconius Genome Consortium 2012) and examples of hybridization promoting adaptation have been found in populations of wall lizards (Yang et al. 2021), cichlids (Meier et al. 2017), and Hawaiian silverswords (Barrier et al. 1999). Hybrid honey bees in the Americas are a mixture of African and European ancestries adapted to tropical and temperate climates. This gives them the capacity to adapt to a wide range of environments across their distribution. The results presented here suggest that adaptation has a polygenic basis, governed by natural selection at loci across the genome, and that this process is likely responsible for adaptation along both latitudinal and elevational clines.

## Materials and Methods

### Sample Collection and Sequencing

We collected worker bees from managed colonies in Carmen de Tonchalá, Norte de Santander, Colombia (320 m above sea level) and Pamplona, Norte de Santander, Colombia (2420 m above sea level) in 2016–2017 (fig. 1). Each sample was collected from a different colony. Newly hatched worker bees were sampled to ensure that they originated in the colony they were collected from. The colonies were located in single apiaries in each location: Carmen de Tonchalá (7°50′43.28″N, 72°34′0.27″W) and Pamplona (7°23′21.86″N, 72°39′1.91″W).

There is very limited movement of managed honey bee colonies in Colombia, and feral colonies are numerous. Beekeepers regularly obtain new colonies from the local landscape, and mating between feral and managed colonies occurs freely. We therefore expect the samples we collected to accurately reflect the genetic ancestry of bees in each locality. DNA was extracted from the thorax of each sample using the Qiagen blood and tissue kit by following the standard protocol. Whole genomic DNA was used to prepare sequencing libraries using the Illumina TruSeq PCR-free kit. All samples were sequenced on an Illumina HiSeqX instrument to produce 2 × 150 bp paired-end reads aiming to obtain mean read coverage >8×.

In addition to the Colombian worker bees sequenced in this study, sequence data were downloaded from the short read archive of National Center for Biotechnology Information (NCBI) for 19 Kenyan worker bees of *A. m. scutellata* for the A reference population (Wallberg et al. 2017), 9 East European worker bees of *A. m. carnica* for the C reference population (Harpur et al. 2014), and 85 Iberian drones of *A. m. iberiensis* for the M reference population (Henriques et al. 2018).

### Mapping and Variant Calling

The raw reads from the Colombian samples and the sequences downloaded from NCBI were mapped to the Amel_HAv3.1 reference genome (Wallberg et al. 2019) using the Burrows–Wheeler alignment tool Burrows-Wheeler Aligner (BWA) version 0.7.17-r1188 (Li and Durbin 2009) with the BWA-MEM algorithm. The Samtools package version 1.14 (Li et al. 2009) was used for sorting the reads on their leftmost coordinates and indexing the BAM-files. The Picard toolkit version 2.18.4 was used to add read groups to the BAM-files (AddOrReplaceReadGroups) and to mark duplicate reads (MarkDuplicates). The new BAM-files were indexed with Samtools as before. Variant calling was done using GATK version 4.0.8.0 following their best practices workflow (https://gatk.broadinstitute.org/hc/en-us/articles/360035535932-Germline-short-variant-discovery-SNPs-Indels-, last accessed 2021-12-05). Filtering of the variants was done using GATK VariantFiltration with the recommended hard filters (https://gatk.broadinstitute.org/hc/en-us/articles/360035890471-Hard-filtering-germline-short-variants, last accessed 2021-12-05) with the limits QD < 2; FS > 60; MQ < 40; MQRankSum < −12.5; ReadPosRankSum < −8; SOR > 3 (QD, QualByDepth; FS, FisherStrand; MQ, RMSMappingQuality; SOR, StrandOddsRatio). Additional filters were applied with VCFtools version 0.1.16 (Danecek et al. 2011) to remove indels and select only bi-allelic sites with a quality value higher than 100 and a maximum average read depth of 20 across all samples (genome-wide mean = 10.3; standard deviation = 5.6; 99.8% of sites have read depth <=20). Sites were also removed if 30% or more of their genotypes were missing in any of the five populations, as those sites would be uninformative.

The 85 Iberian drones, which are haploid, were treated as diploid samples during variant calling. After the filtering steps mentioned above, 7.1 million SNPs remained and out of those, 178,000 SNPs (2.5%) were heterozygous in one or more of the drones. At sites where only one drone was heterozygous, the genotype of this individual was marked as missing. The 38,500 sites where more than one drone was heterozygous were removed from the entire dataset (0.5% of the total number of SNPs). An additional filter for minor allele count greater than or equal to three was also applied with VCFtools, leaving a final set of 5.4 million SNPs. One of the Iberian drones had unexpectedly high heterozygosity after diploid variant calling, with an inbreeding coefficient of *F* = 0.26 compared with an average value of *F* = 0.98 for all other drones (calculated with VCFtools and the –het option). As the data from this individual was regarded as unreliable, it was excluded from the analyses.

### Identification of Centromeres

We estimated the location of pericentromeric regions, characterized by low GC (guanine + cytosine) content, in the Amel_HAv3.1 genome by identifying regions with low GC content over extended chromosomal segments. GC content was calculated in 10 kbp windows based on the reference genome, except in windows where no bases were called. To identify low GC regions, a cumulative window-based score was used. Each 10 kbp window was given a score +1 if its GC content was lower than the genome-wide average, −1 if it was higher than the genome-wide average, and 0 if it was equal to the genome-wide average. The cumulative score was calculated from left to right on each chromosome, starting at zero and adding the value from each window. The 0.5 Mb region with the highest increase in the cumulative score on each chromosome was then selected as the starting point for the putative centromere. This region was then extended in both directions, by ten windows at a time, if the score increased with at least five within those ten windows. Finally, the region was extended by one window at a time if this window increased the score by 1. Extended low GC regions identified in this way showed high correspondence with those regions identified in a previous study (Wallberg et al. 2019) based on a previous version of the genome (Amel_HAv3; see supplementary figs. S8–S11, Supplementary Material online and supplementary table S4, Supplementary Material online).

### Genetic Variation

The nucleotide diversity (π) was calculated with VCFtools, separately for each of the reference populations and the Colombian populations. The calculations were done in 50 kbp windows across the genome before computing the average of those windows. The filtering of the VCF (Variant Call Format) files described above includes a filter for minor allele count, which in this case could cause biased estimates, as rare SNPs are excluded. Therefore, a set of 7 million SNPs, including all allele counts but otherwise filtered as described above, was used instead. Pairs of haploid Iberian drone samples were randomly combined into pseudodiploid samples to conduct this analysis. We assessed the samples for evidence of relatedness using the –relatedness2 option in VCFtools (Manichaikul et al. 2010; Danecek et al. 2011).

### Neighbor-Joining Tree and PCA-plot

We used a thinned set of 372,205 SNPs to produce a neighbor-joining tree and PCA-plot. This was obtained from the filtered set of 5.4 million SNPs by thinning with VCFtools so that the minimum distance between any pair of SNPs was 500 bp. For the tree, a pairwise distance matrix was calculated with VCF2Dis (https://github.com/BGI-shenzhen/VCF2Dis). The tree was constructed in R version 4.1.2 (R core team 2021) with the package ape version 5.5 (Paradis et al. 2004) and the neighbor-joining algorithm (Saitou and Nei 1987). The PCA was performed with plink v1.90b4.9 (Purcell et al. 2007) and the eigenvectors were plotted in R.

### Analysis of Genome-Wide Ancestry

The genome-wide ancestry of all 142 individuals (19 Kenyan A group bees, 9 East European C group bees, 85 Iberian M group bees, and 29 Colombian admixed bees) was estimated with the clustering software ADMIXTURE (Alexander et al. 2009) on the same thinned SNP-set that was used for the tree and PCA. VCFtools and plink were used to convert the VCF-file to the .ped and .map files required by ADMIXTURE. In order to treat the drones as haploid samples, they were specified as male in the .ped file and then the option –haploid=“male:*” was used when running ADMIXTURE. In ADMIXTURE, the number of clusters to be formed is specified by the parameter K. As the expected number of ancestral clusters in this case is three, ADMIXTURE was run with K-values ranging between two and seven, performing unsupervised clustering with 10-fold CV. The calculations for all K-values were repeated in eight independent runs with different seeds and the major modes of the results were identified using the software pong version 1.5 (Behr et al. 2016).

### *F_ST_*-based Scans for Differentiation and Peak Identification

The pairwise divergence between populations was analyzed with *F_ST_* (Weir and Cockerham 1984) on the set of 5.4 million SNPs obtained after filtering. The calculations were done in 10 kbp windows (or in some cases 1 kbp windows) across the genome using VCFtools version 0.1.16 (Danecek et al. 2011) with the option –weir-fst-pop. Three different *F_ST_*-comparisons were made: the Colombian highland population versus the Colombian lowland population and each of the Colombian populations separately versus the Kenyan samples.

Peaks were identified based on 10 kbp windows with *F_ST_* greater than or equal to the 0.998 quantile of all *F_ST_*-values. In order to generate a more comprehensive list of candidate genes we also extended the peak coordinates into neighboring flanking regions with elevated *F_ST._* The regions were extended in each direction if the adjacent window had *F_ST_* greater than or equal to the 0.98 quantile of all *F_ST_*-values, as long as the total mean *F_ST_* of the peak (weighted by the number of SNPs per window) was greater than or equal to the 0.99 quantile of all *F_ST_*-values. Genes for which at least 30% of the exons overlap the peak by at least 50% of the exon length were identified as potential targets of selection. We estimated recombination rate of the most prominent peaks by comparison to an linkage-disequilibirum-based recombination map (Wallberg et al. 2015).

### Coalescent Simulations

We performed coalescent simulations and compared patterns of divergence between simulated and empirical data using ms (Hudson 2002), taking an approach similar to (Wallberg et al. 2017). In our model, an ancestral population would split into a highland and a lowland population, respectively, with unchanged and constant population sizes and without subsequent gene flow between them. The two populations would evolve to reach the same average *F*_ST_ as our empirical data. At this stage, we would sample multiple independent SNPs and compare the *F*_ST_ distributions between simulated and empirical SNPs, and test for enrichment of high-*F*_ST_ SNPs in the empirical data. To parameterize the simulation, we filtered the 5.4 million SNPs to contain only those that were polymorphic in the 29 Colombian bees (4.8 million). For this data, we estimated the population mutation rate of Watterson's theta (*θ*_W_; i.e., the number of segregating sites) (Watterson 1975) to 0.47%/bp across the genome. Linkage disequilibrium decays within a couple of hundred base-pairs in the honey bee (Wallberg et al. 2015), and we therefore took the thinned SNP dataset (372,205 SNPs; 582 bp per SNP) to represent the empirical distribution of divergence across independent loci. We filtered also this dataset to be polymorphic in Colombian bees, producing 248,598 SNPs (906 bp per SNP). Using these SNPs, we estimated the genome-wide *F*_ST_ between the two populations using Reynolds *F*_ST_ estimator (Reynolds et al. 1983) to 0.0584. We converted *F*_ST_ into the scaled time parameter *T* = 0.0301 using the equation *T* = –ln (1 – *F*_ST_)/2, and used T/2 in the simulation (0.0150). Using these parameters, we simulated a 906 bp long locus with the scaled population mutation rate of 4.23 (906 × 0.00467). We ran this simulation 248,598 times, exporting one biallelic SNP and sampling 28 highland and 30 lowland copies of the SNP locus per iteration. The ms command was:

ms 58 248598 -t 4.23 -I 2 28 30 -ej 0.0150 2 1 -s 1 > highland_vs_lowland.simulated.txt

From both the simulated and empirical datasets, we computed and compared allele frequencies and per-SNP Weir-Cockerham *F*_ST_ values (Weir and Cockerham 1984). In addition, we produced length-matched (906 bp) window-based *F*_ST_ estimates across the genome and determined the 0.998 quantile. The 0.998 quantile at this fine resolution is 0.255, close to the 10 kbp quantile of 0.21 in the empirical data.

### Homology and Gene Ontology Enrichment

To test for shared biological functions among the highly divergent genes, we used protein-protein BLAST (Camacho et al. 2009) in order to detect homology between RefSeq honey bee protein sequences and *Drosophila* sequences from FlyBase (version dmel_r6.38_FB2021_0) (Larkin et al. 2021), taking the highest scoring fly gene as the honey bee homolog. We then used VCFtools (Danecek et al. 2011) and the honey bee gene coordinates to compute the average weighted *F*_ST_ per gene. We tested for enriched gene ontology terms among genes with *F*_ST_ surpassing the 0.998 quantile in ShinyGO (Ge et al. 2020), using the *Drosophila* homologs for the top set as the foreground (*n* = 14) and all homologs in the set as background (*n* = 9,818) and a False Discovery Rate cutoff of 0.05 to correct for multiple testing. ShinyGO calculates the significance of enrichment based on the hypergeometric distribution followed by false discovery rate correction.

### Analysis of Clinal Variation in Ancestry

Calfee et al. (2020) fitted ancestry clines across hybrid zones in Argentina and California, where honey bees transition from predominantly African (A) to predominantly European (M and C) ancestry. The clines were constructed for each SNP with a logistic model and their steepnesses reflect the rates of type A ancestry change. As the range of A ancestry in the Argentinian populations (2%–76%) partly overlaps with the range of A ancestry in our Colombian populations (68.6%–84.7%), while the Californian populations all have lower A ancestries (<50%), we made comparisons to the Argentinian cline steepness estimates.

Out of the set of 542,000 SNPs analyzed by Calfee et al., 394,000 SNPs (72.6%) were also included in our SNP-set. Those SNPs were analyzed in a similar way as in the study by Calfee et al. in order to get ancestry probabilities that could be compared to the clines across Argentina. The software ancestry_HMM (Corbett-Detig and Nielsen 2017) was used to estimate the posterior probability of each ancestry type (A, C, and M) for each SNP and each individual. In order to make the input to the program as similar as possible to what was used by Calfee et al., allele frequencies from the same reference populations were used instead of the reference populations used in other parts of this study. The genetic distances between the markers were copied from the SNP-set used by Calfee et al., either directly or as the sum of distances between intermediate markers which were not included in our SNP-set. The allelic depths for each of the Colombian individuals were extracted from the VCF-file and the genome-wide ancestry estimated by ADMIXTURE was used as the prior ancestry estimate for each population. The admixture scenario modeled was an initial population of the C group, which was first subject to a migration pulse from the M group and then more recently admixed with the A group. The time of both pulses was estimated between 2 and 150 generations before present, with a prior estimate of 100 generations for the first pulse and 60 generations for the second pulse. The effective population size was set to 670,000 (Nelson et al. 2017). The posterior probabilities calculated by the program for each SNP and each possible combination of ancestries (C + C, C + M, C + A, M + M, M + A, A + A) were recalculated to the total probability of A ancestry per SNP as p(A) = p(AA) + (p(MA) + p(CA))/2. Finally, the average A ancestry per SNP was calculated for each population. The difference between the A ancestry in the Colombian highland population and the Colombian lowland population was calculated as lowland ancestry proportion minus highland ancestry proportion. Then the Pearson correlation between those ancestry differences and the Argentinian cline steepness estimates was calculated across the genome.

The Pearson correlation of the A ancestry per SNP was also calculated between the Colombian highland and lowland populations and each of the Argentinian populations separately. For each of the Argentinian populations, the difference between the correlation to the Colombian highland population and the Colombian lowland population was evaluated with the method suggested by (Hittner et al. 2003) for comparing two overlapping correlations based on dependent groups, using the R package cocor v. 1.1-4 (Diedenhofen and Musch 2015).

## Supplementary Material

evad157_Supplementary_Data

## Data Availability

Previously published data used in this study were collected from NCBI Sequence Read Archive (SRA) from BioProject PRJNA893245, PRJNA216922, and PRJNA357367. Data produced by this study has been deposited at NCBI SRA at PRJNA892909.
