# Supplementary material for: The Genomic Basis of Adaptation to High Elevations in Africanized Honey Bees
Source: Genome Biol Evol. 2023 Aug 25;15(9):evad157. doi: 10.1093/gbe/evad157 (PMC10484329; doi:10.1093/gbe/evad157)
Supplement: evad157_Supplementary_Data [file evad157_supplementary_data.zip › Supplementary Information Legends.docx]

Supplementary Figures

Figure S1

Left: PCA of Colombian highland samples (blue) and lowland samples (red), showing the two first components, based on 372,205 SNPs.

Right: Eigenvalue of each principal component.

Figure S2 – S4:

*F_ST_* in 1 kbp windows across chromosome 11. The *F_ST_* -values in the regions defined as peak 11a and peak 11b (see Results section and Table S1-S2) as well as in the mTOR gene are marked in colour (cyan = peak 11a; orange = peak 11b; black = mTOR). The magenta-coloured bar shows the location of the region identified by Nelson et al. (2017). The quantile corresponding to 99.8% of the *F_ST_* -values is shown as a dashed yellow line. The shaded grey area marks the putative pericentromeric region. S1: *F_ST_* of Colombian highland population versus Kenyan samples; S2: *F_ST_* of Colombian lowland population versus Kenyan samples; S3: *F_ST_* of Colombian highland population versus Colombian lowland population.

Figure S5 – S7:

*F_ST_* in 1 kbp windows across chromosome 14. The *F_ST_* -values in the region defined as peak 14a (with coordinates from the Colombian highland versus lowland *F_ST_* -scan; see Results section and Table S3) are coloured in green. The quantile corresponding to 99.8% of the *F_ST_* -values is shown as a dashed yellow line. Shaded grey area marks the putative pericentromeric region. S4: *F_ST_* of Colombian highland population versus Kenyan samples; S5: *F_ST_* of Colombian lowland population versus Kenyan samples; S6: *F_ST_* of Colombian highland population versus Colombian lowland population

Figure S8-S11:

GC-content in 10 kb windows and putative pericentromeric regions per chromosome, based on the reference genome Amel_HAv3.1. Gray graph = cumulative window score, as defined in Methods; brown points = GC content per window; yellow dashed line = mean genomic GC content; red triangles = fraction of missing data per window; blue dashed line = putative pericentromeric region based on window score; green line = pericentromeric region identified in a previous version of the reference genome (Amel_HAv3, Wallberg *et al.* 2019).

Supplementary Tables:

Table S1:

Metadata and read coverage for all samples included in this study.

Table S2:

Peaks identified in *F_ST_* -scans. Chromosomal coordinates, number of SNPs and *F_ST_* (mean *F_ST_* from 10 kbp windows weighted by the number of SNPs in each window) given for each peak in each *F_ST_* -comparison (HL = Colombian highland population; LL = Colombian lowland population; Kenya = Kenyan samples (A group))

Table S3:

List of genes found in each of the regions of elevated *F_ST_* found in the three *F_ST_* comparisons.

Table S4:

Coordinates and mean GC-content of putative pericentromeric regions on each chromosome.

Table S5:

*F_ST_* values between the highland and lowland populations for every gene.

Table S6:

Enriched GO terms for genes with high *F_ST_* between the highland and lowland population.
