# Supplementary figures and images for: The Genomic Basis of Adaptation to High Elevations in Africanized Honey Bees
Source: Genome Biol Evol. 2023 Aug 25;15(9):evad157. doi: 10.1093/gbe/evad157 (PMC10484329; doi:10.1093/gbe/evad157)

Figure S1

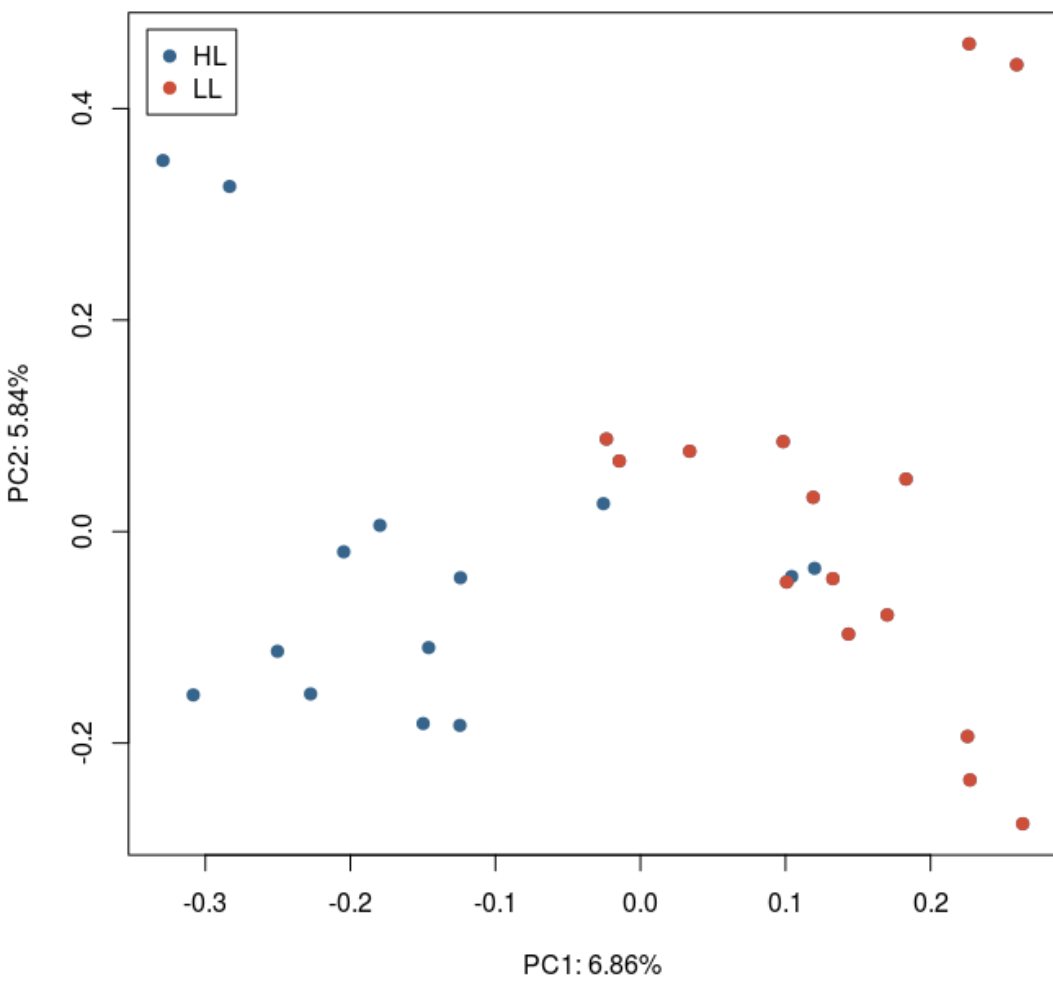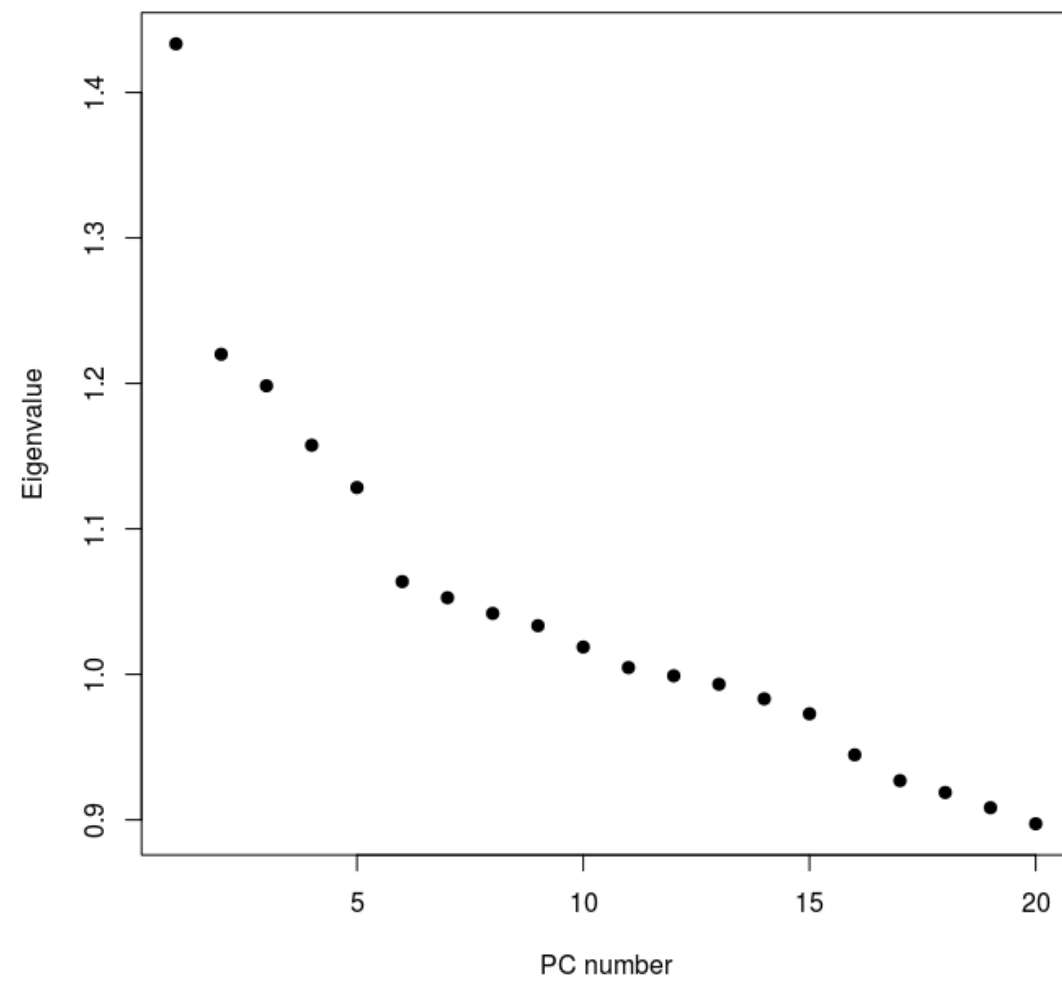

Figure S2

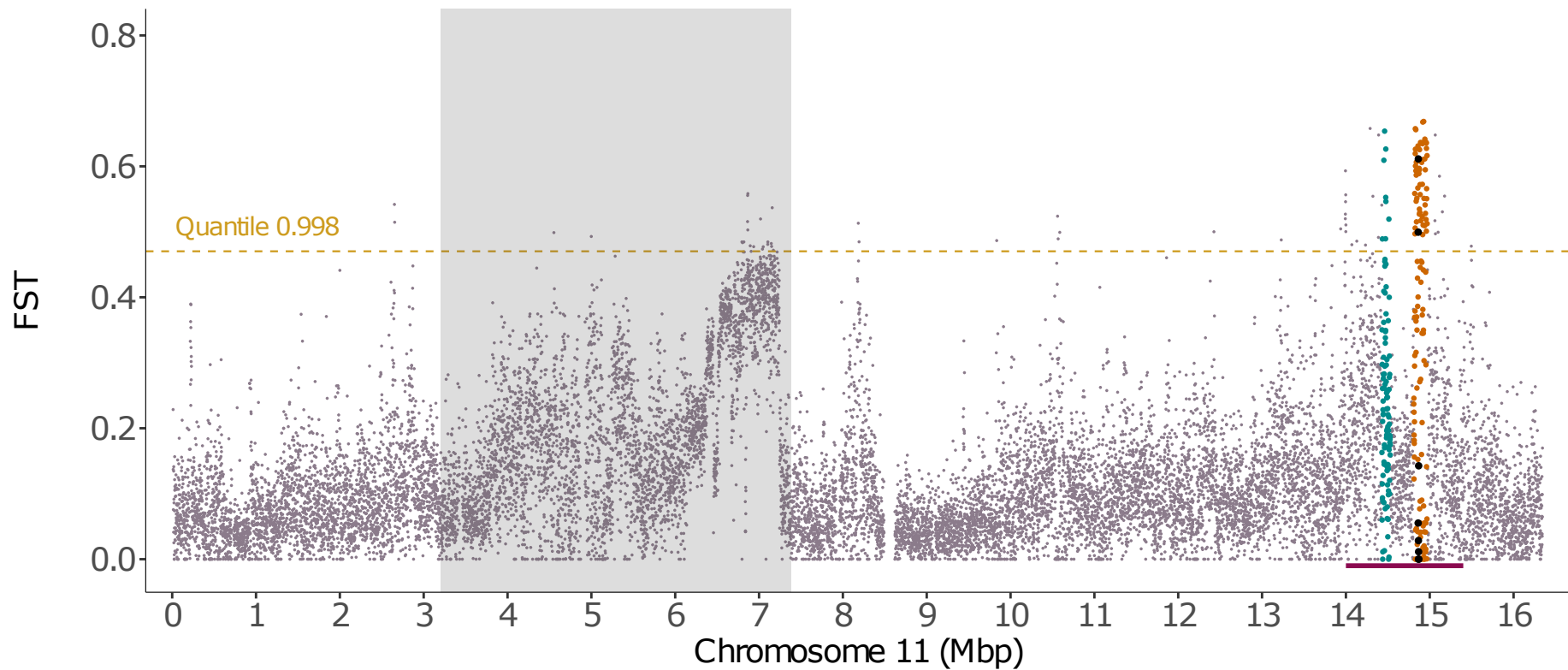

Figure S3

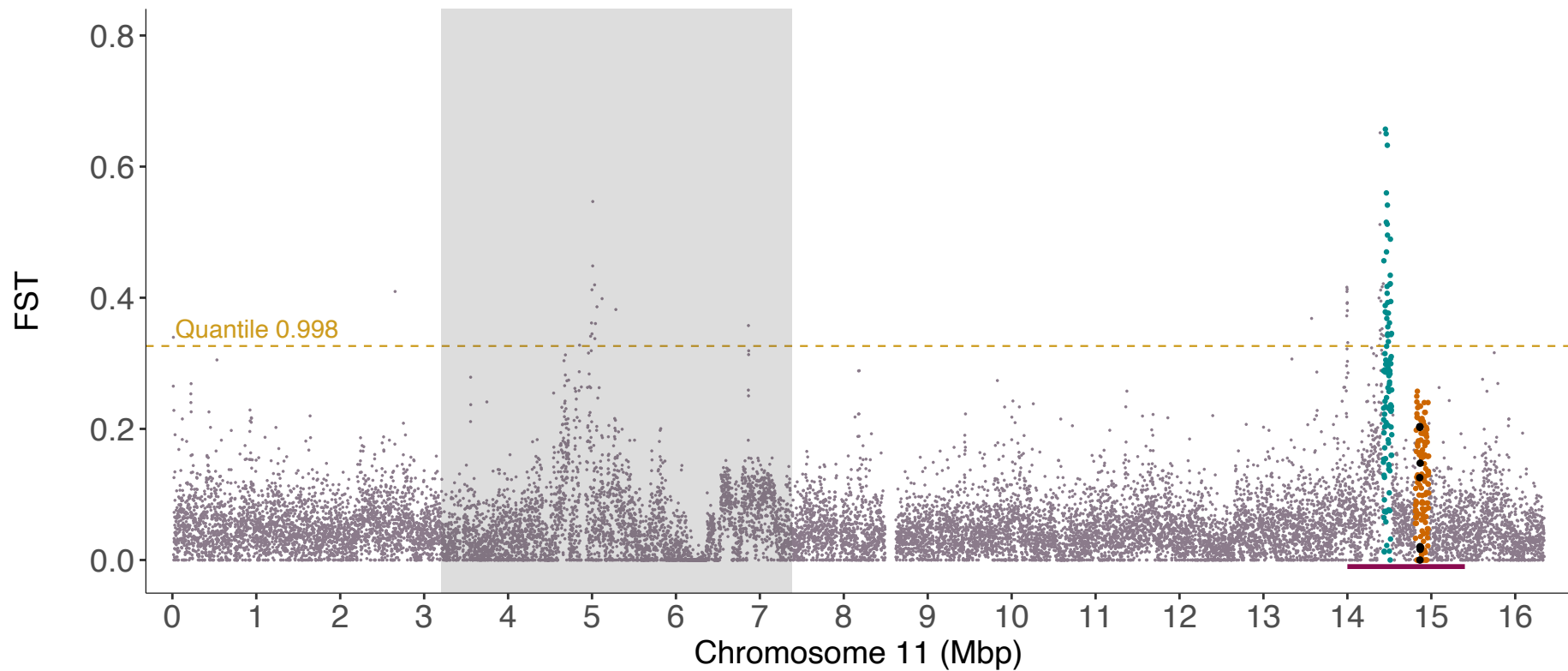

Figure S4

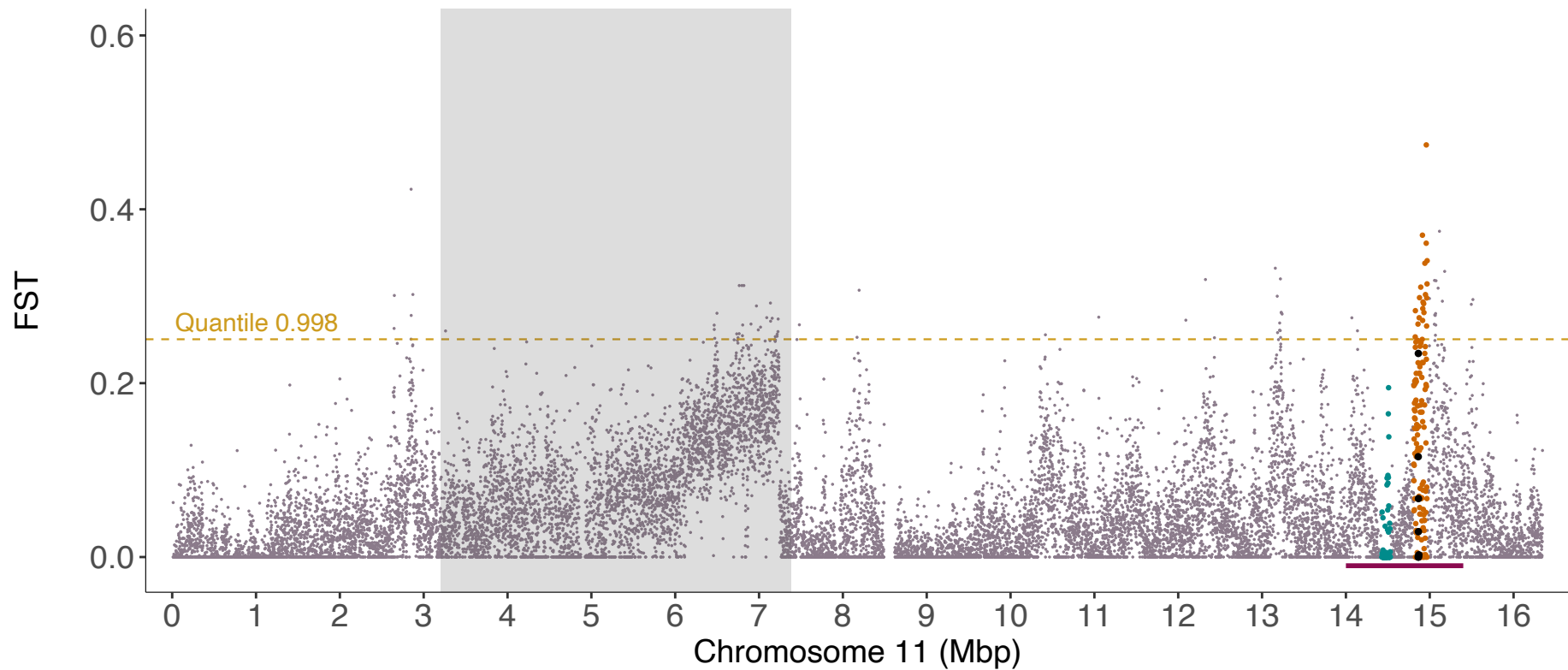

Figure S5

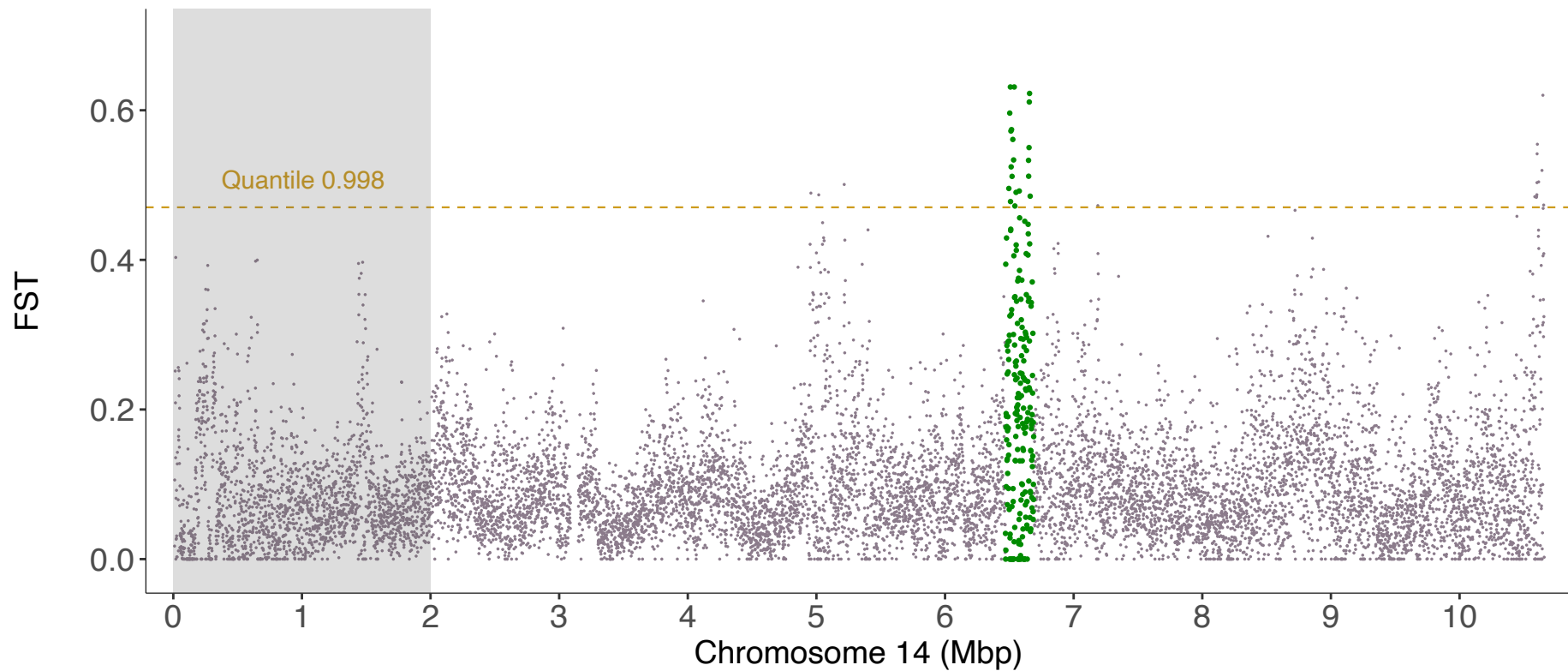

Figure S6

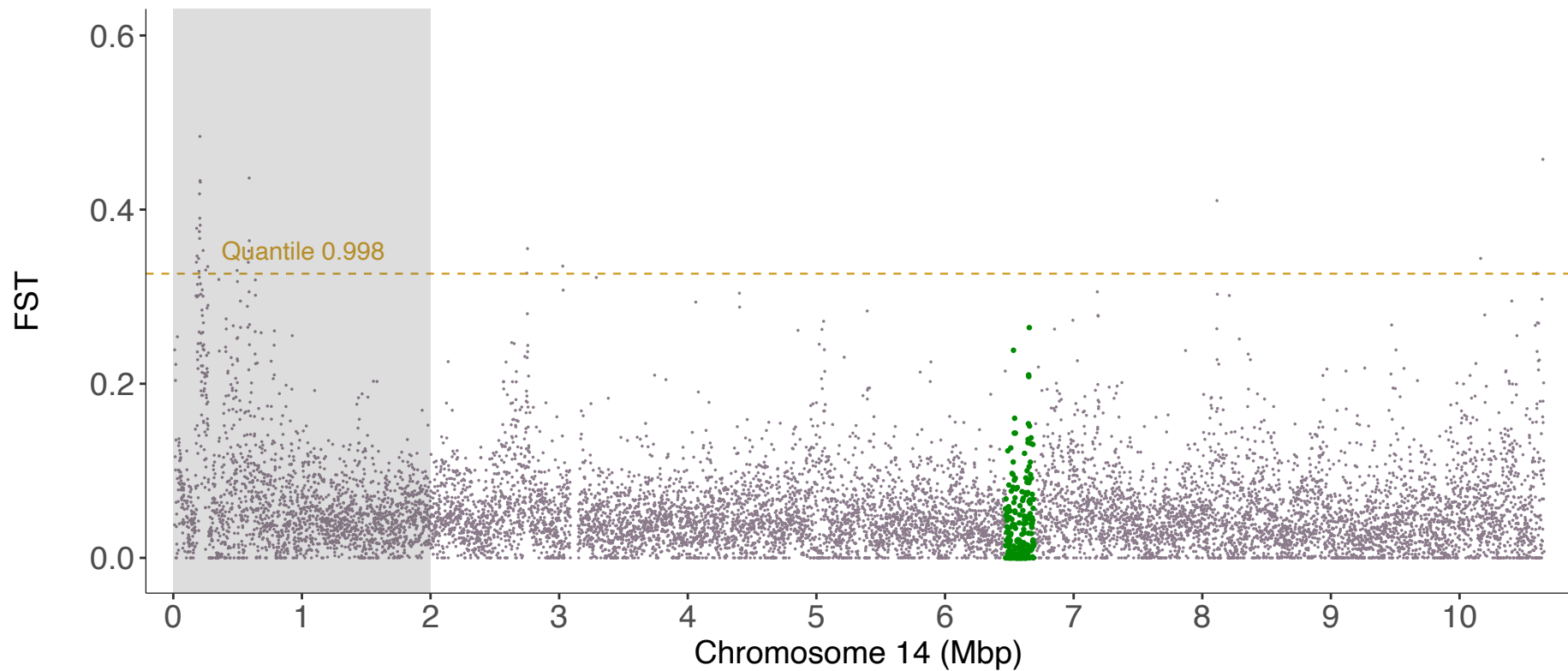

Figure S7

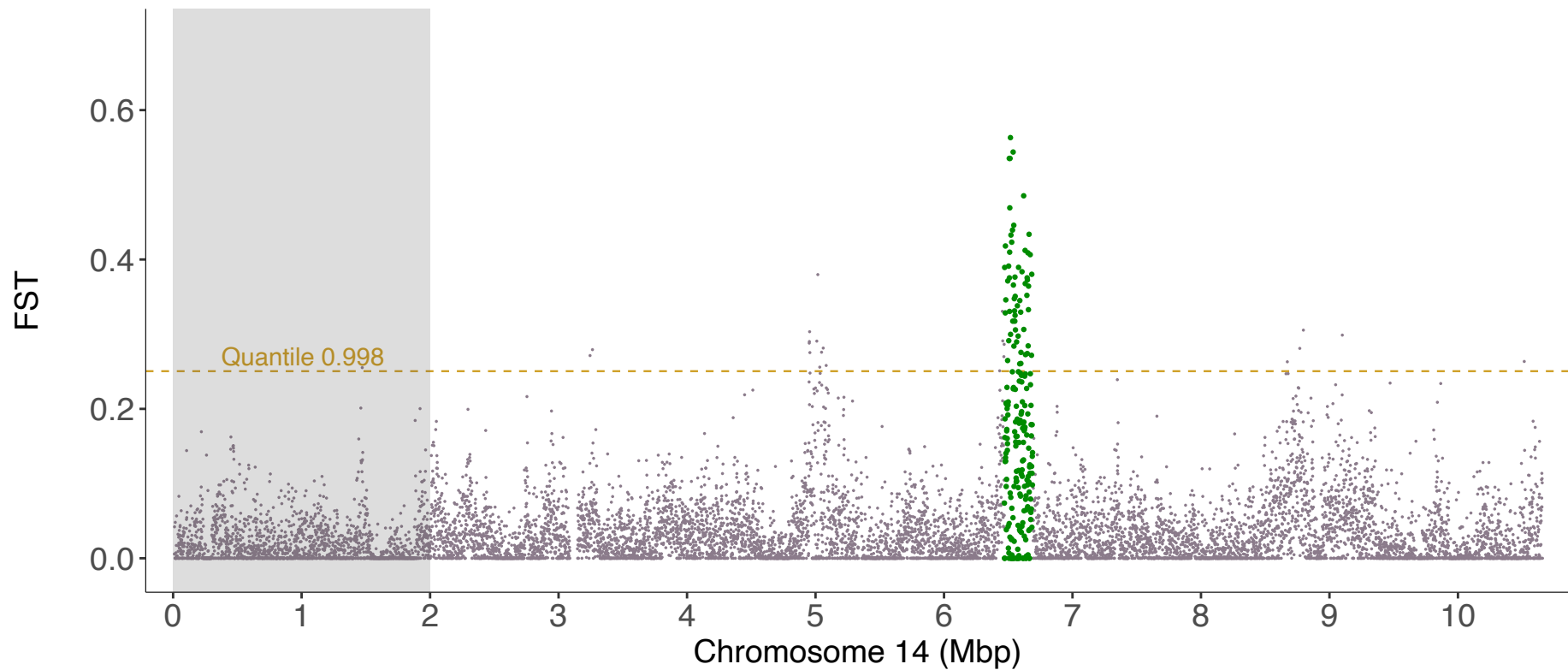

Figure S8

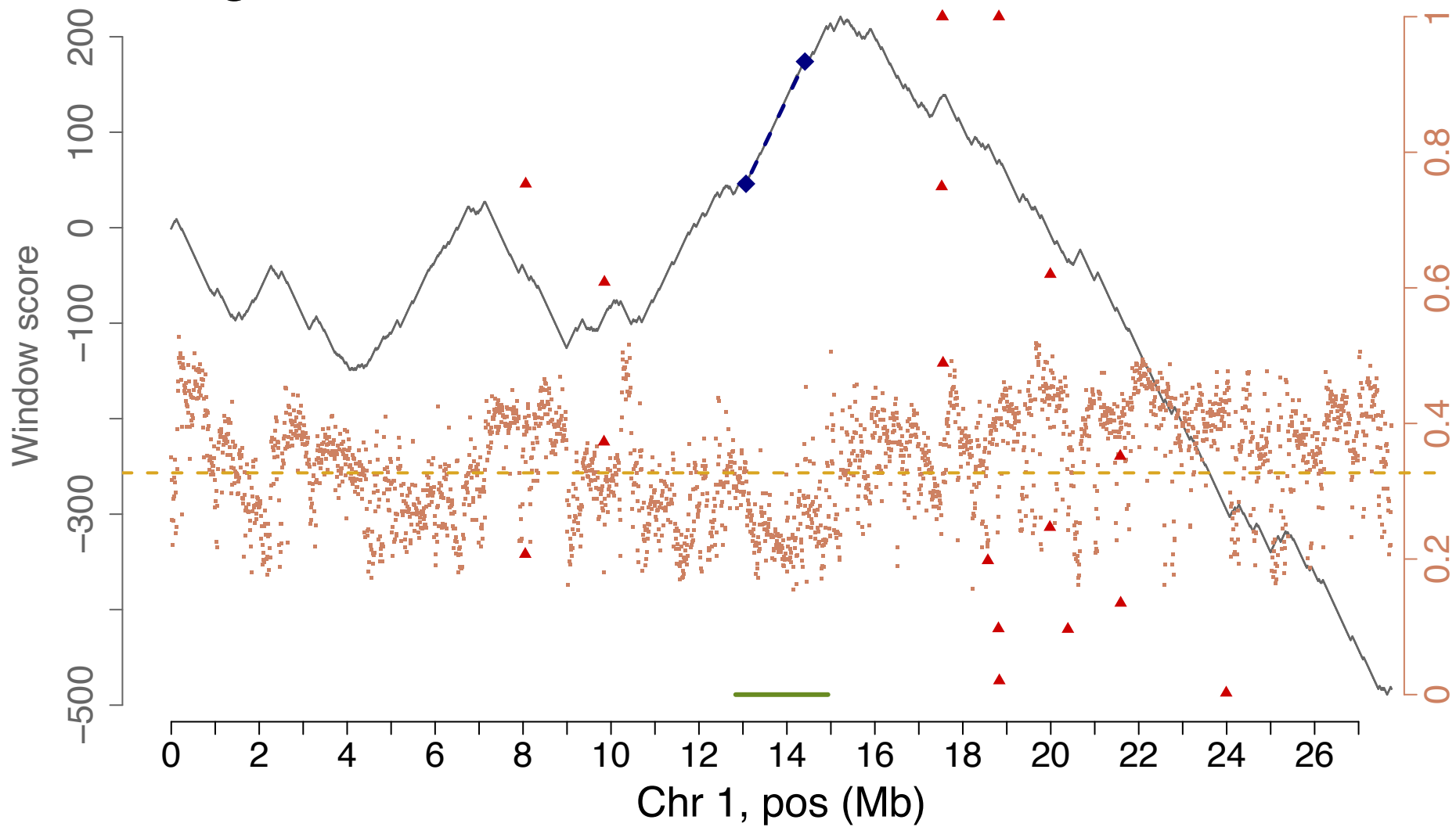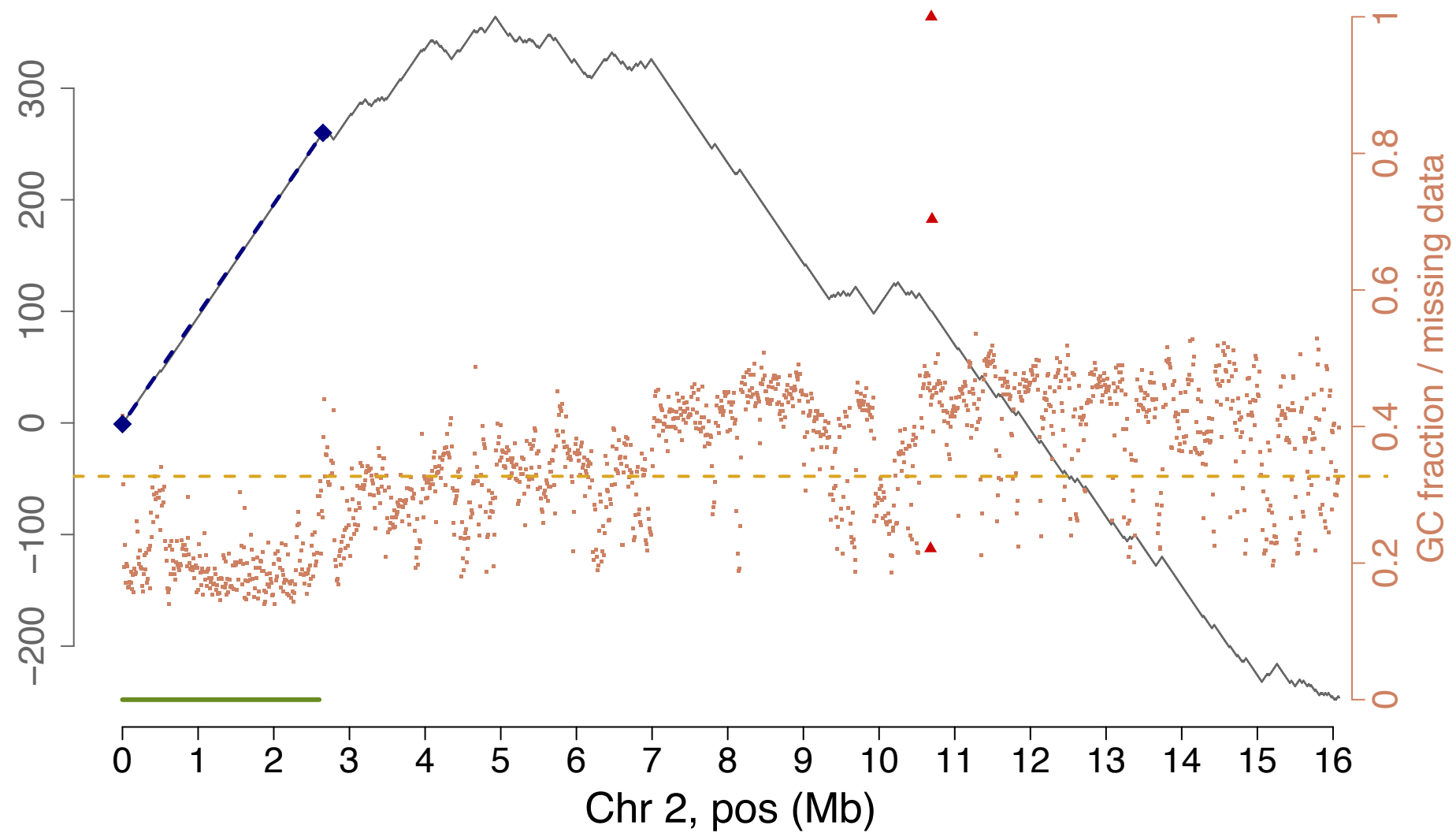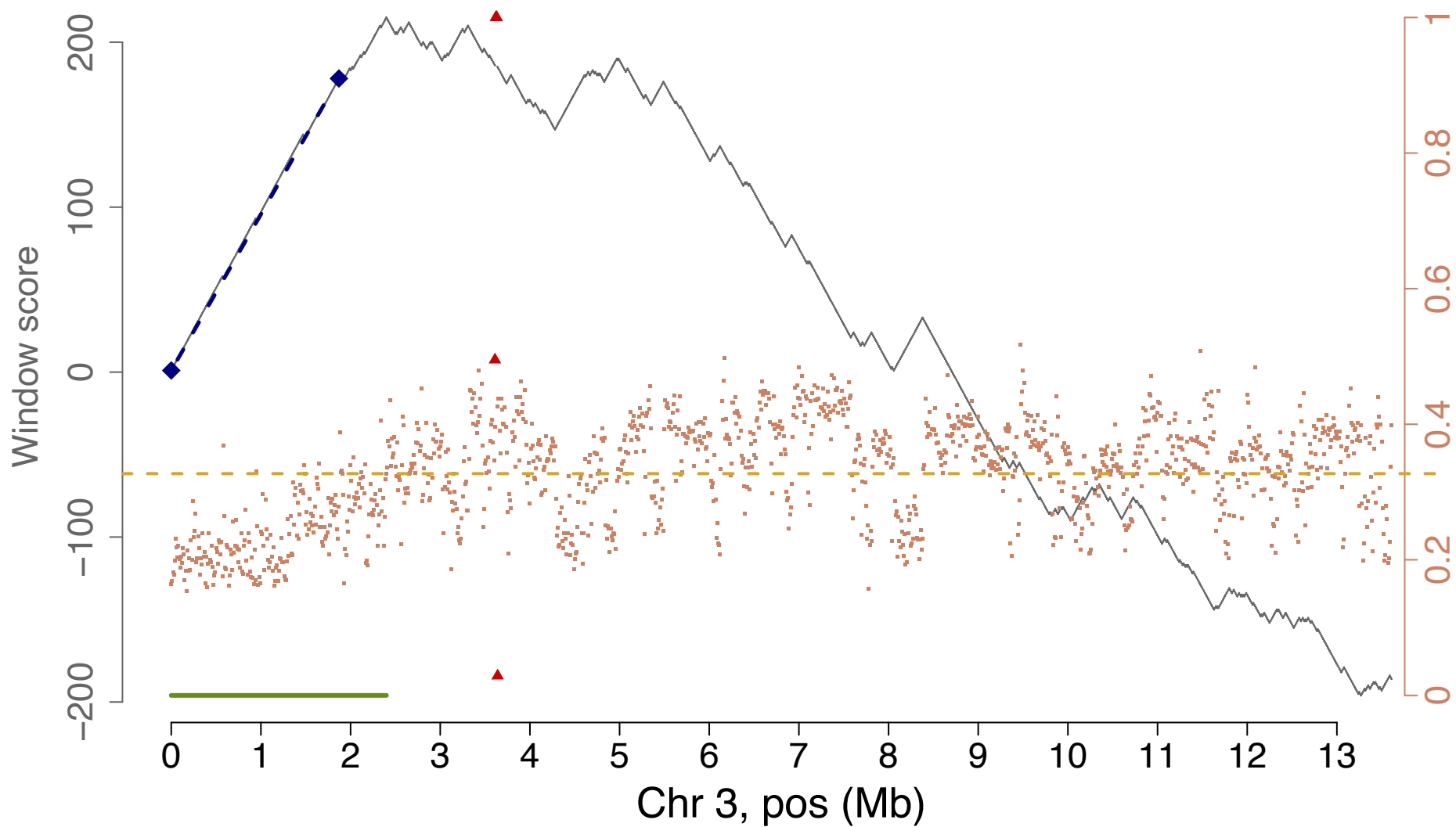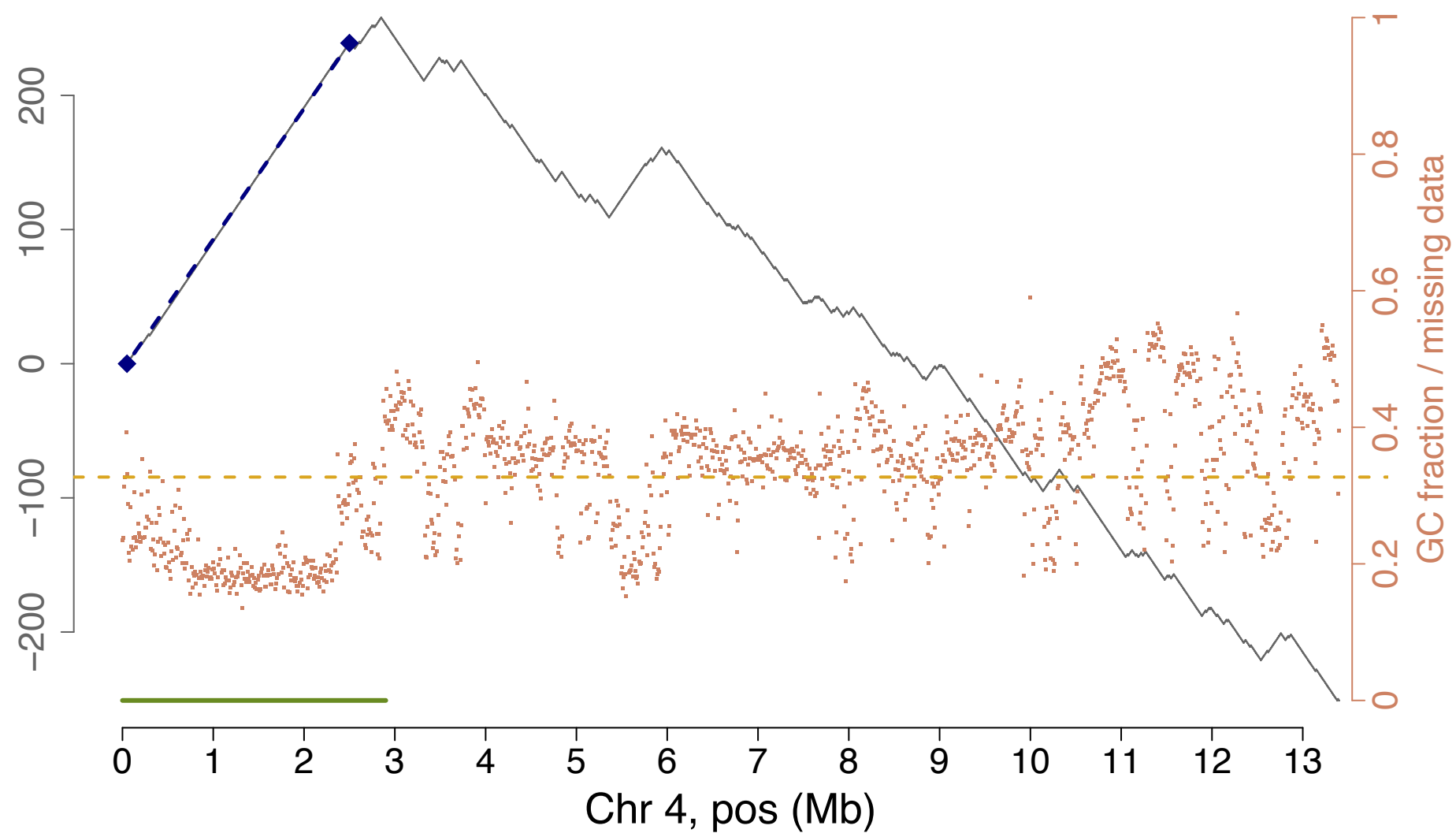

Figure S9

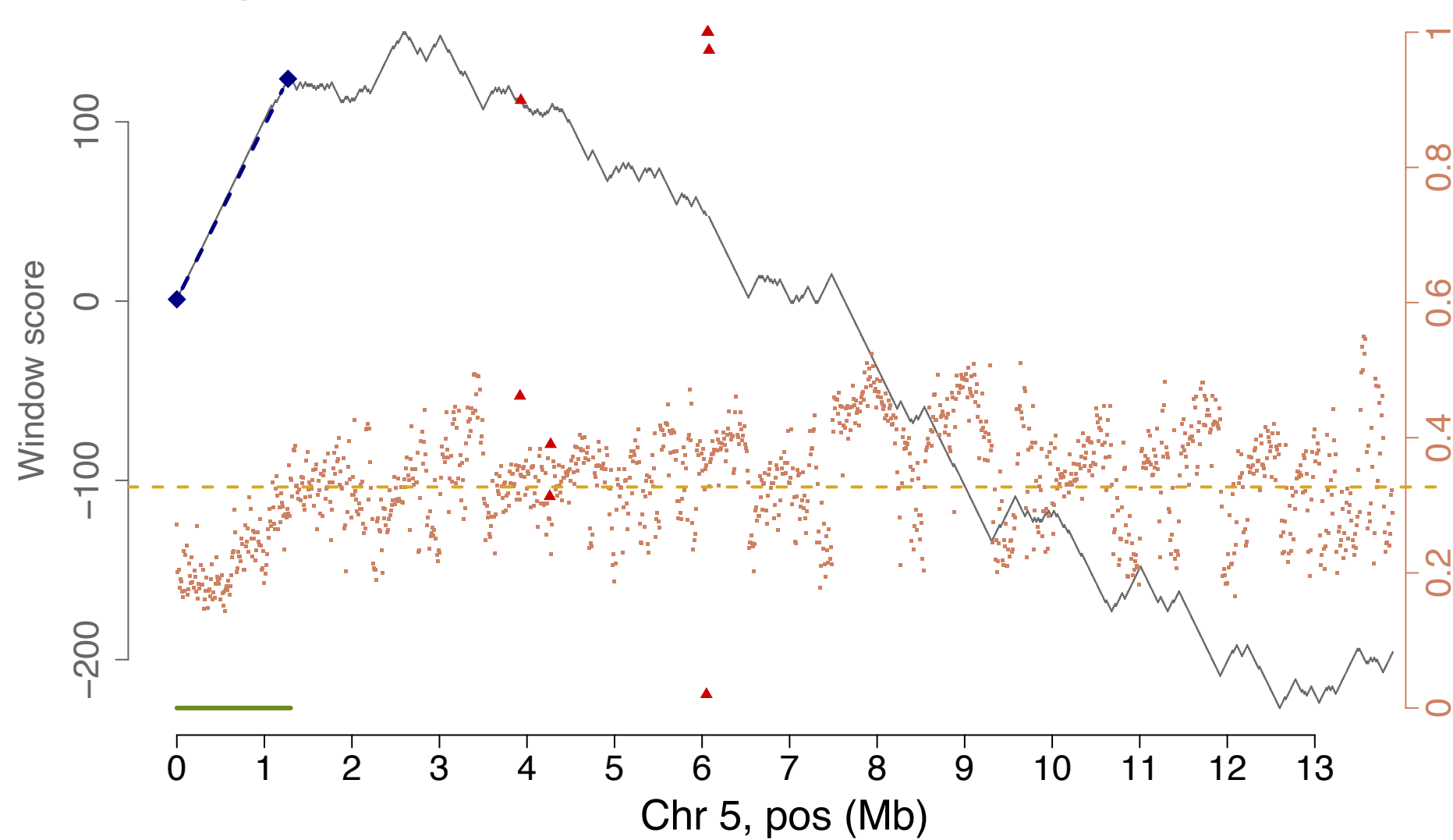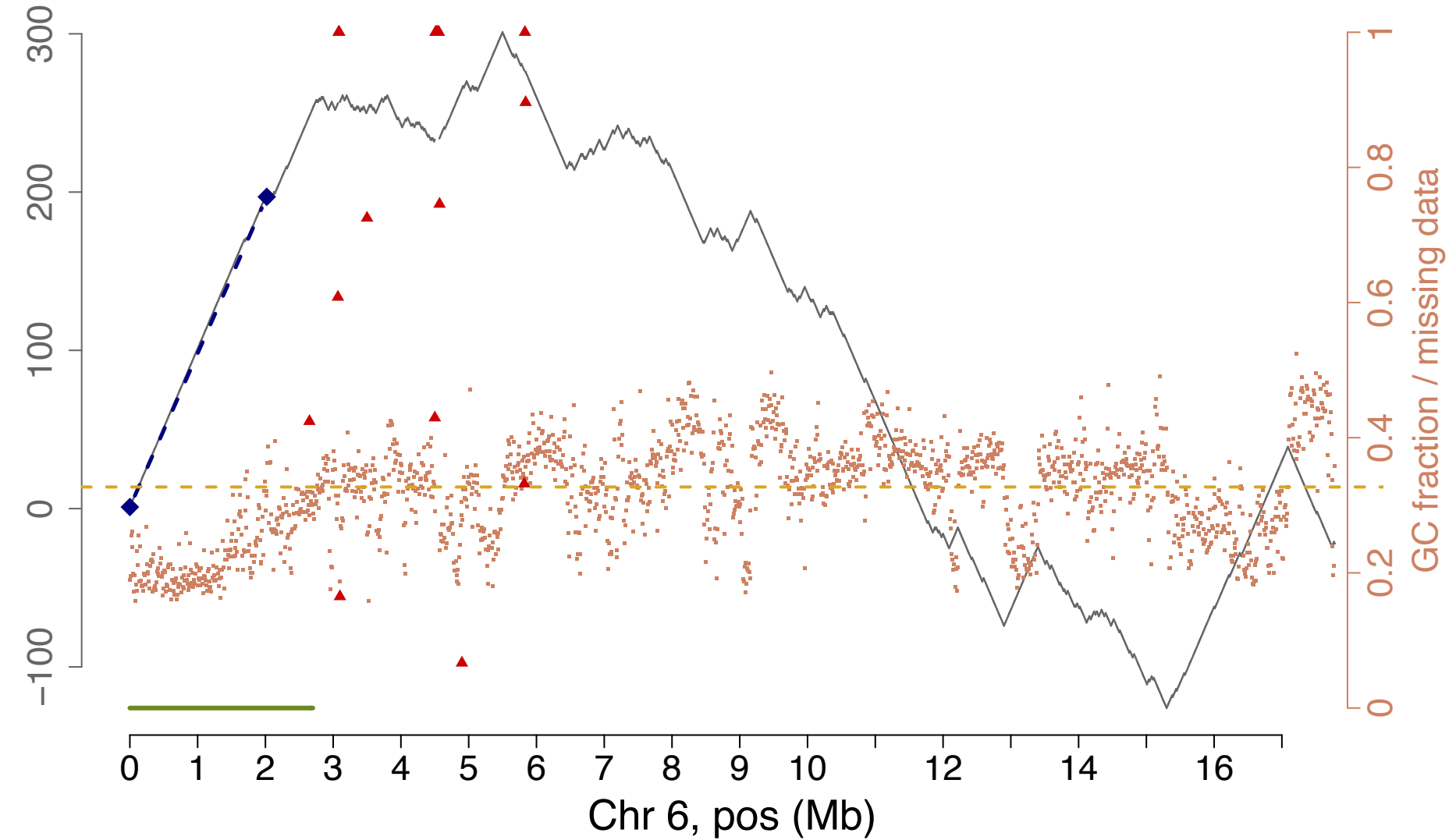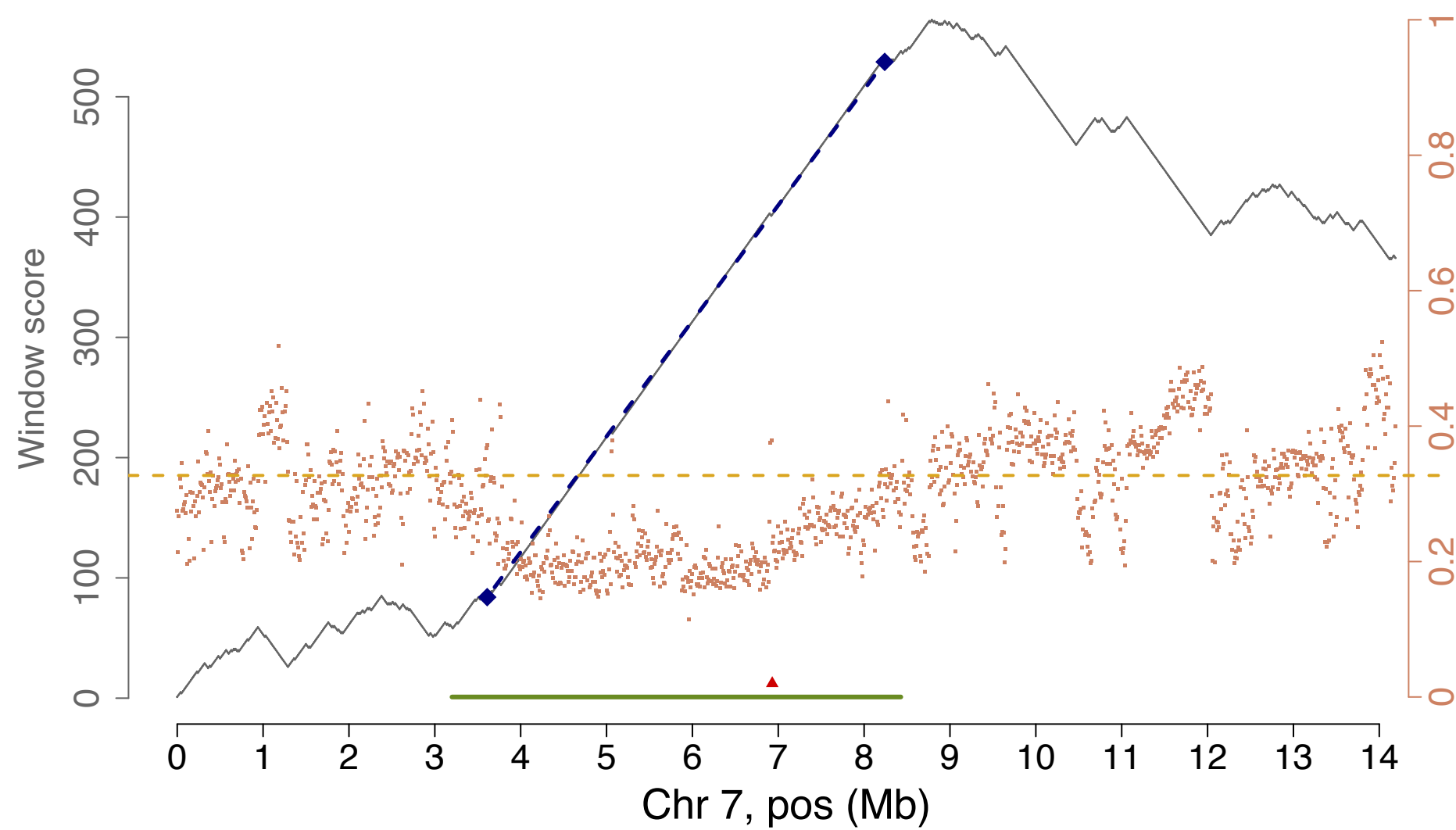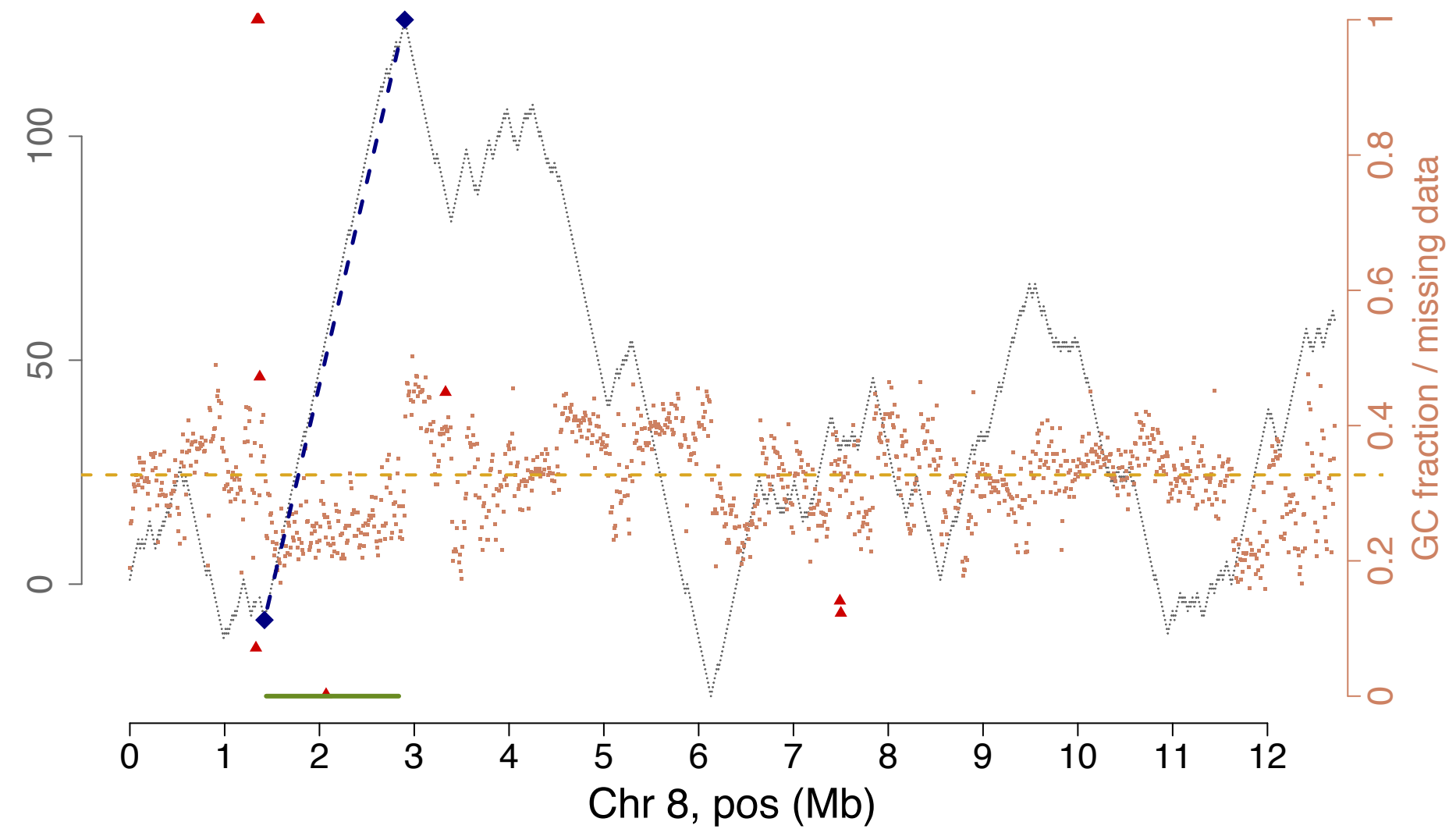

Figure S10

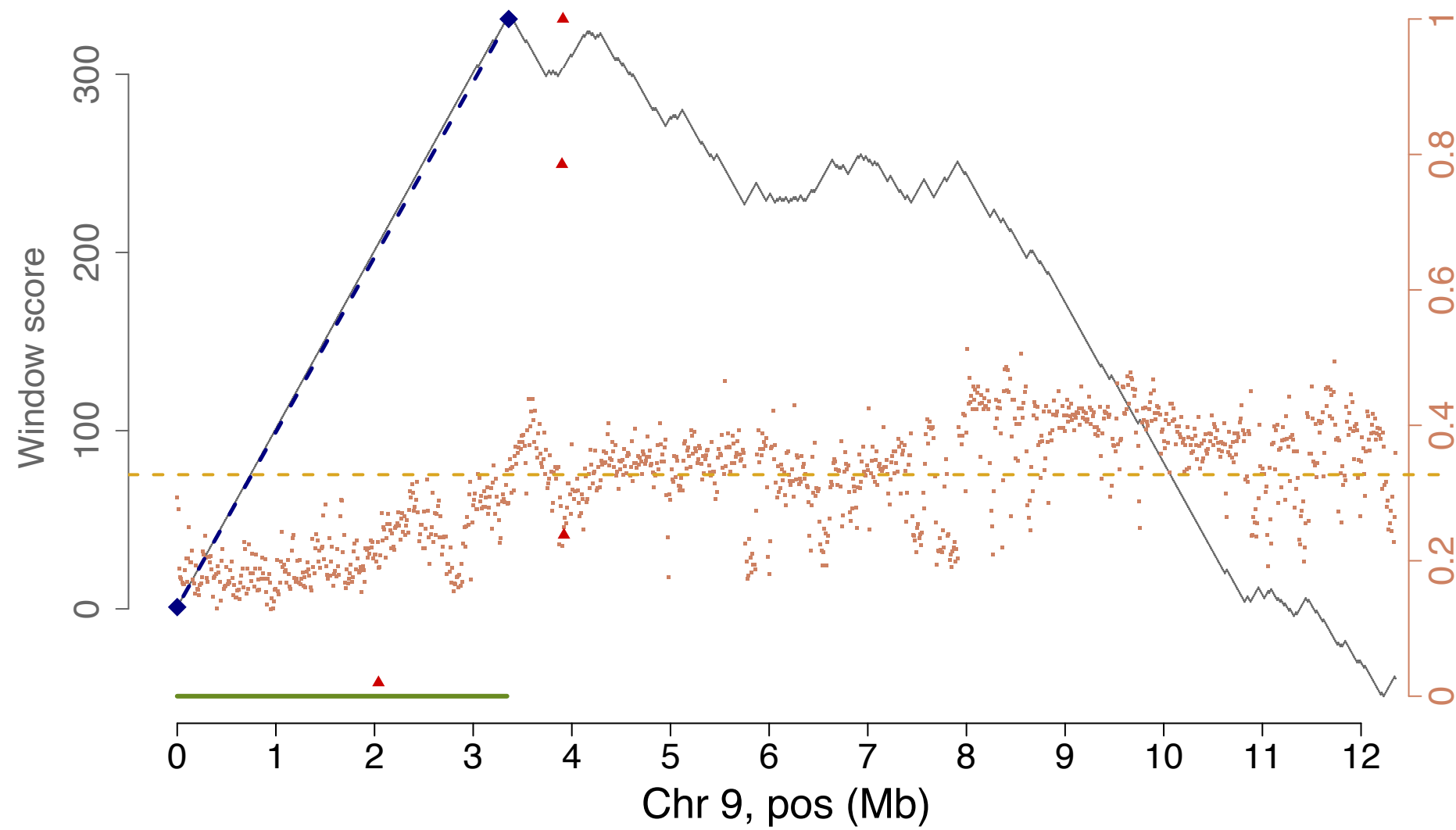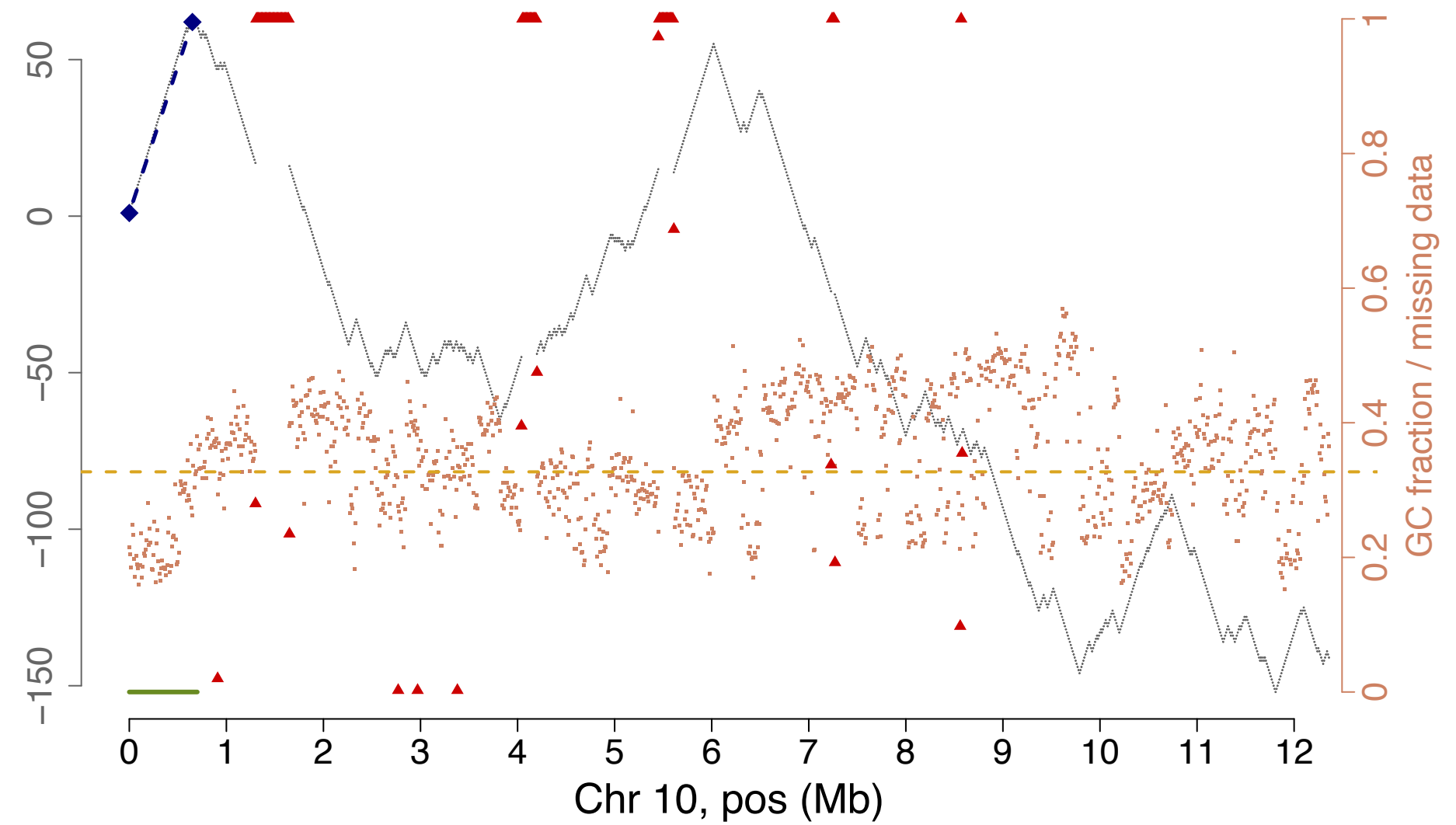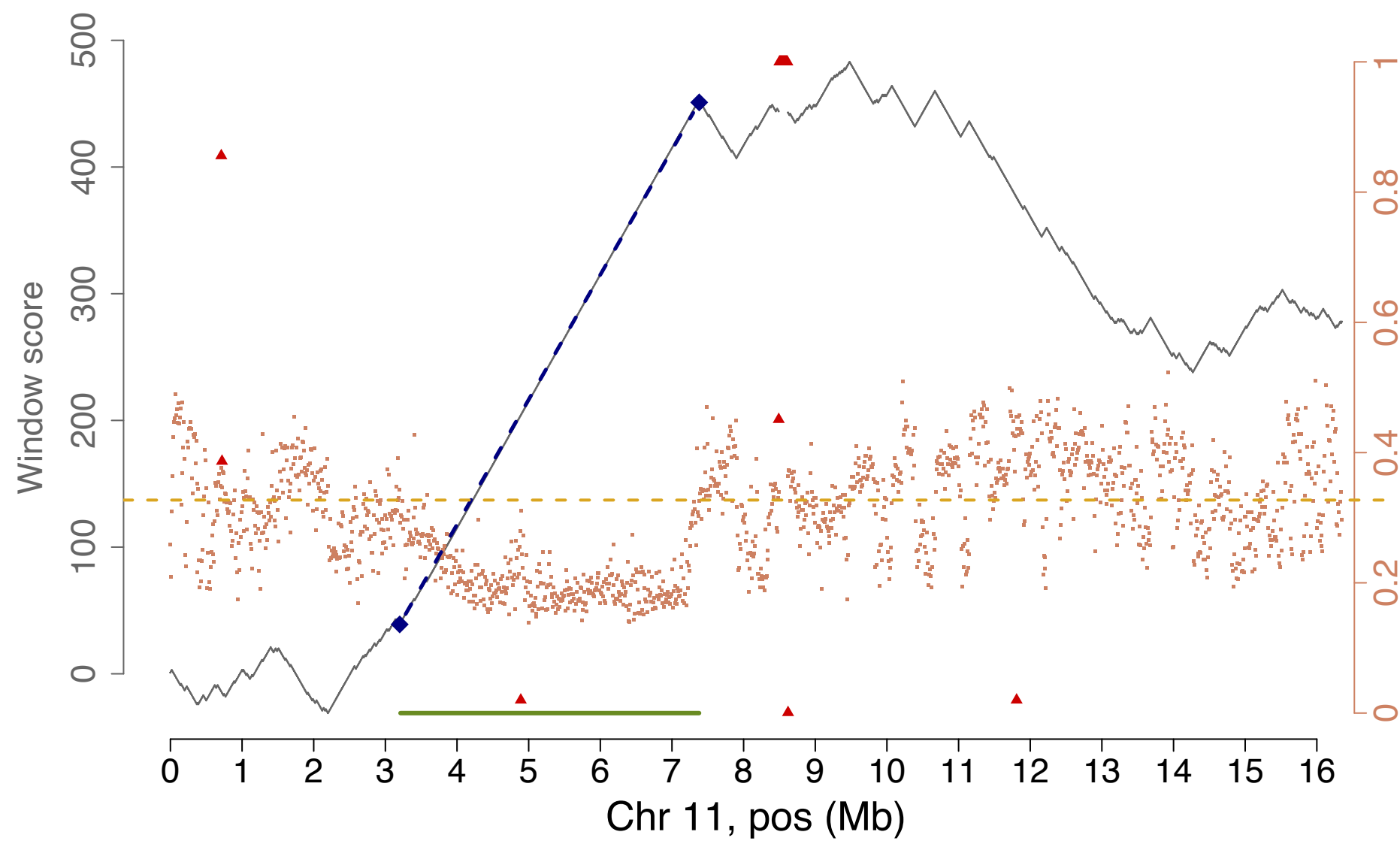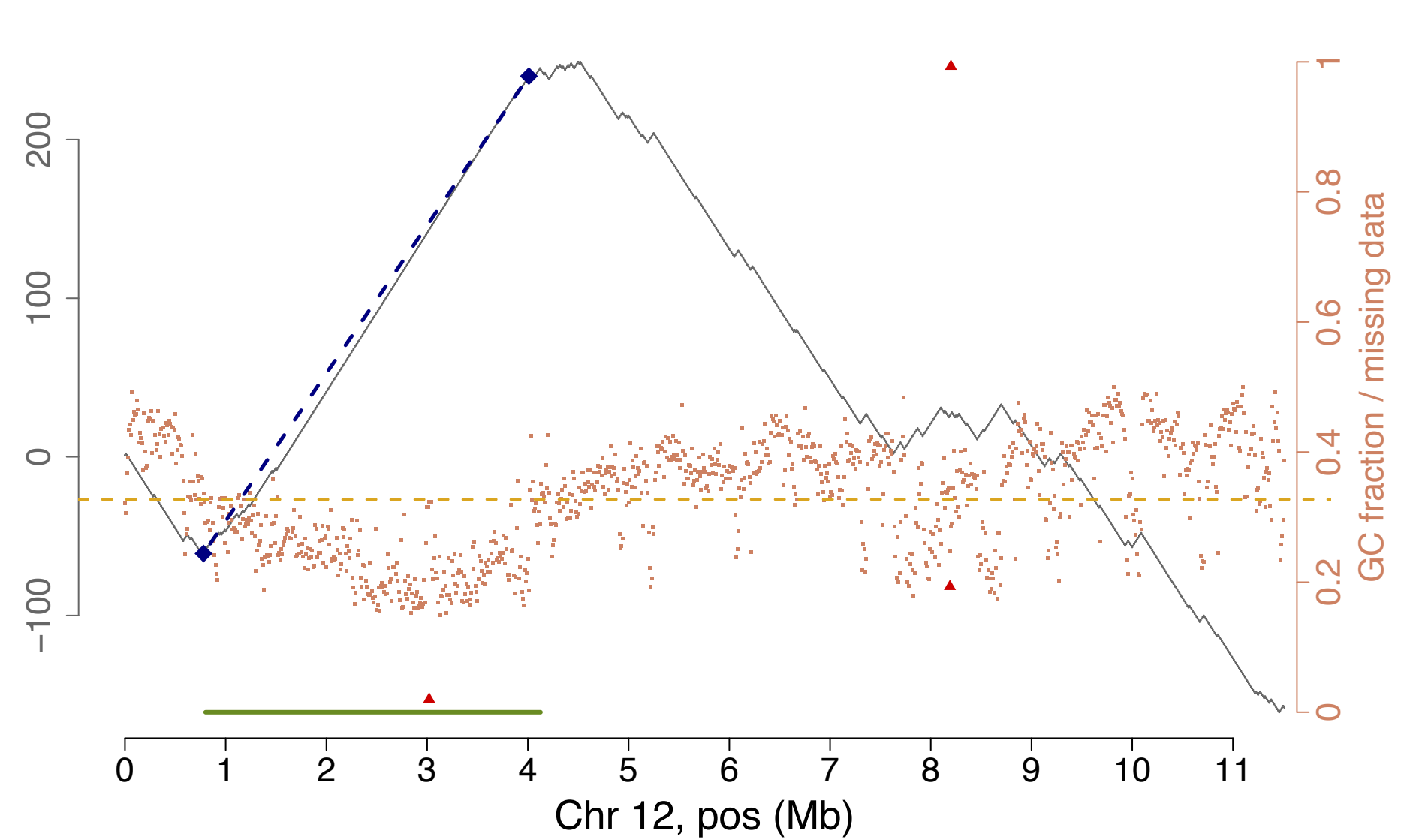

Figure S11

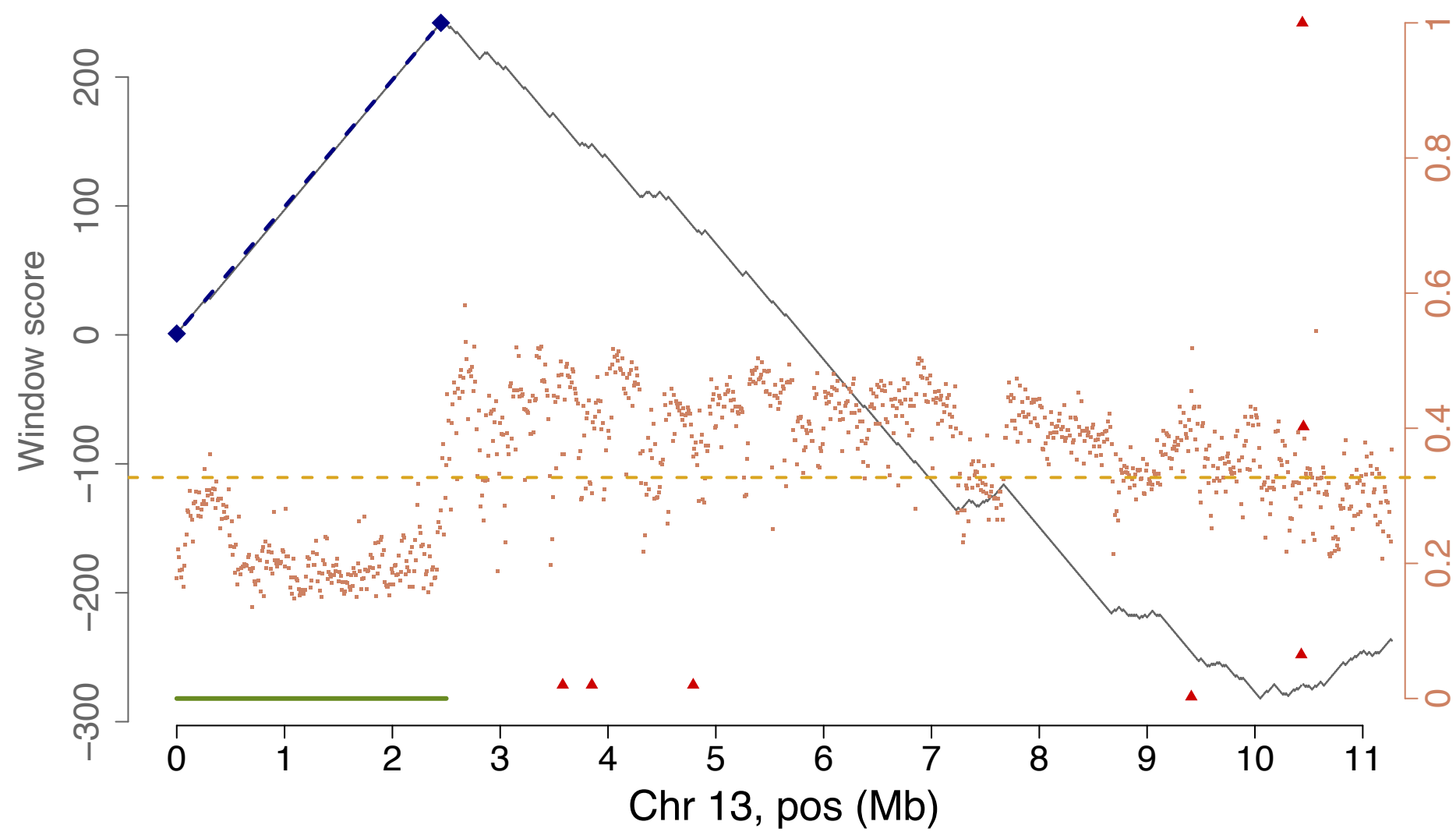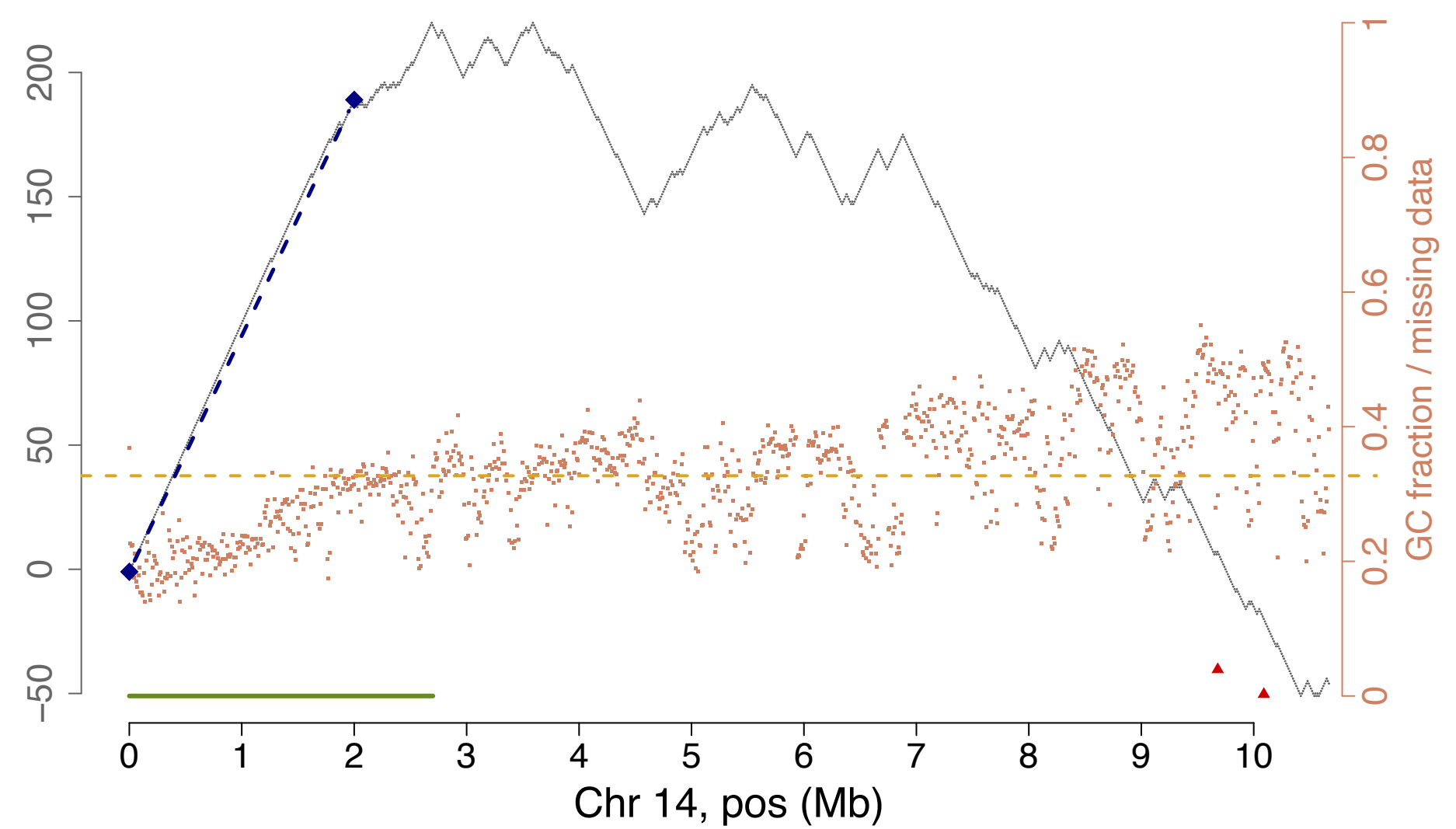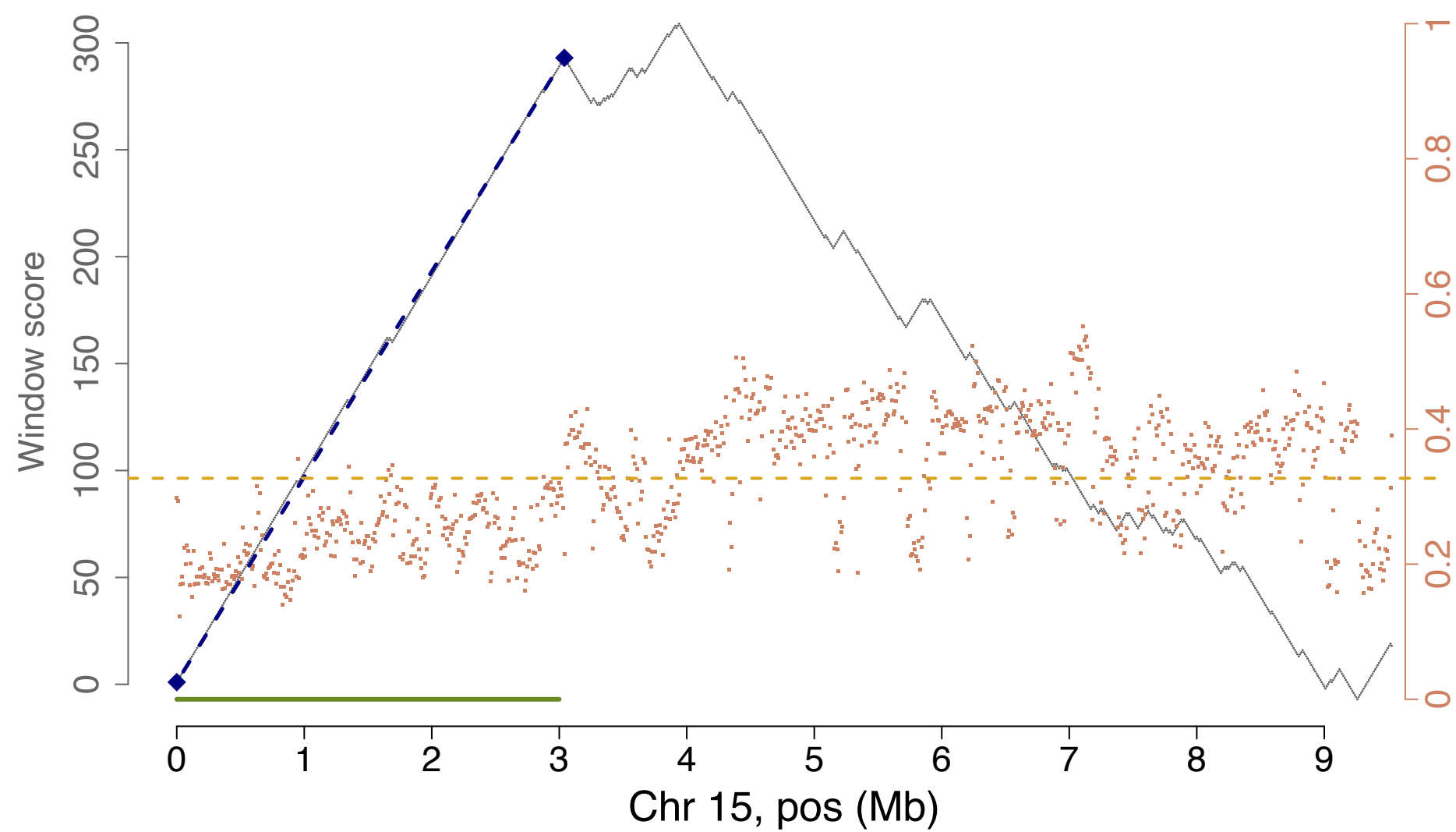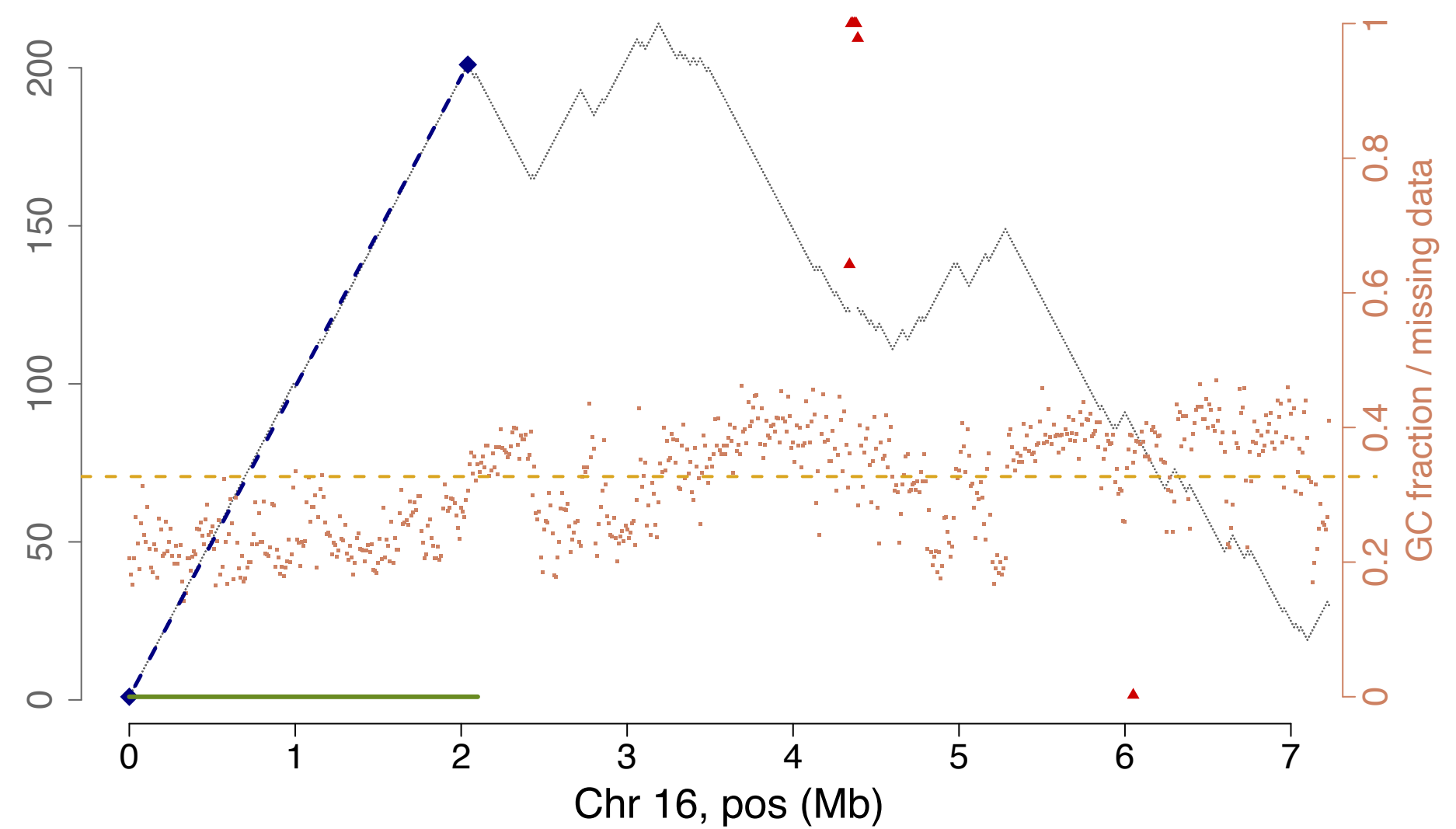

Supplement: evad157_Supplementary_Data [file evad157_supplementary_data.zip › suppfigs_everittV3.pdf]
